# Supplementary material for: Biosynthesis of thiocarboxylic acid-containing natural products
Source: Nat Commun. 2018 Jun 18;9:2362. doi: 10.1038/s41467-018-04747-y (PMC6006322; doi:10.1038/s41467-018-04747-y)
Supplement: Supplementary file 1 — Supplementary Information [file 41467_2018_4747_MOESM1_ESM.pdf]

Supplementary Information

Biosynthesis of thiocarboxylic acid-containing natural products

Dong *et al.*

Supplementary Methods

General experimental procedures. All ^1H, ^{13}C, and 2D NMR (^1H-^1H COSY, ^1H-^{13}C HSQC, ^1H-^{13}C HMBC, ^1H-^1H ROESY) experiments were run on a Bruker Avance III Ultrashield 700 at 700 MHz for ^1H and 175 MHz for ^{13}C nuclei. Preparative HPLC was carried out on an Agilent 1260 Infinity LC equipped with an Agilent Eclipse XDB-C18 column (250 mm × 21.2 mm, 7 μm). LC-MS was performed on an Agilent 1260 Infinity LC coupled to a 6230 TOF (HRESI) equipped with an Agilent Poroshell 120 EC-C18 column (50 mm × 4.6 mm, 2.7 μm). Optical rotations were obtained using an AUTOPOL IV automatic polarimeter (Rudolph Research Analytical). UV was measured with a NanoDrop 2000C spectrophotometer (Thermo Scientific). IR spectra were attained using a Spectrum One FT-IR spectrophotometer (PerkinElmer).

Culture conditions. *E. coli* strains harboring plasmids or cosmids were grown in lysogeny broth (LB) with appropriate antibiotic selection. *Streptomyces* strains were grown on solid ISP4 or MS media at 28 °C or cultured in liquid tryptic soy broth (TSB) at 28 °C and 250 rpm, with appropriate antibiotic selection, if needed. *E. coli-Streptomyces* conjugations were plated onto ISP4 or MS media supplemented with 10 mM $MgCl_2$. Fermentation of *S. platensis* recombinant strains were conducted as below. Briefly, fresh spores of *Streptomyces* strains were inoculated into TSB seed medium and cultured for 2 days. PTM fermentation medium (dextrin, 40 g, lactose, 40 g, yeast extract, 5 g, MOPS, 20 g, trace elements, 10 ml, in 1 L water, pH 7.3) was inoculated with 4% (v/v) seed culture and 3% (w/v) Amberlite XAD-16 resin (Sigma-Aldrich) and incubated at 28 °C and 250 rpm for 7 days[1].

Extraction and LC-MS analysis. Extraction of resin from small-scale fermentations followed previously reported protocols[1]. After fermentation of the recombinant *Streptomyces* strains, the resin was harvested by centrifugation, washed three times with H_2O, and extracted three times with CH_3OH. Liquid chromatography for LC-MS analysis was performed using an 18 min solvent gradient (0.4 mL min^{-1}) from 5% – 100% either CH_3CN or CH_3OH in H_2O containing 0.1% formic acid. For the detection of high-polarity small molecules (i.e. ADHBA and ADHBSH), the fermentation broth (aqueous layer) was directly used for LC-MS analysis and

eluted using a 10 min solvent gradient (0.4 mL min^{-1}) from 0% – 10% CH_3CN in H_2O containing 0.1% formic acid.

Analytical size-exclusion chromatography. The molecular weights (MW) and quaternary state of PtmA3 and PtmU4, respectively, in solution were determined by size-exclusion chromatography using a Superdex 200 16/600 column (GE Healthcare Life Sciences) connected to an ÄKTAxpress system (GE Healthcare LifeSciences). The column was pre-equilibrated with two column volumes of 50 mM Tris buffer, pH 8.0, and calibrated with ribonuclease A (13.7 kDa), carbonic anhydrase (29 kDa), ovalbumin (44 kDa), conalbumin (75 kDa), aldolase (158 kDa), and ferritin (440 kDa). The chromatography was carried out at 4 °C at a flow rate of 1 mL min^{-1}. The calibration curve of K_{av} versus log(MW) was prepared using the equation $K_{av} = (V_e - V_o) / (V_t - V_o)$, where V_e, V_o, and V_t is the elution volume, column void volume, and total bed volume, respectively.

Protein secondary structure analysis by circular dichroism. Circular dichroism (CD) was measured on a Jasco J-815 Circular Dichroism Spectropolarimeter. Data were collected at 1.0 nm intervals in the wavelength range of 180−290 nm with a scanning speed of 50 nm min^{-1} at 20 °C. The proteins were diluted to 0.3 mg mL^{-1} using CD buffer (10 mM potassium phosphate, 100 mM potassium fluoride). A 0.1 cm cuvette containing 140 µL of protein sample was used for all the measurements. Measurements were performed in triplicate with buffer background subtracted from each sample spectrum. The far UV CD spectra (200−240 nm) were selected and analyzed using the K2D3 method for protein secondary structure prediction[2].

Antibacterial assay. ThioPTM (**1**) and thioPTN (**2**) were tested for antibacterial activities against *Staphylococcus aureus* ATCC 25923 and *Kocuria rhizophila* ATCC 9431. The minimum inhibitory concentration (MIC) values were determined using a 96-well plate format with Muller-Hinton (MH) broth[3]. Briefly, 2 µL of each compound, serially diluted in DMSO, were added to wells containing 98 µL of cell cultures diluted to an OD_{600} = 0.005. The MIC values, performed in triplicate, were determined after incubation at 37 °C for 18 h. PTM (**3**) and PTN (**4**), and DMSO were used as positive and negative controls, respectively.

Phylogenetic Analysis in Figure 3. Protein sequences of one- and two-domain type III CoA transferases were obtained from the NCBI database: 739U4 (*Streptomyces platensis* CB00739; AIW55577), 765U4 (*S. platensis* CB00765; AIW55622), 7327U4 (*S. platensis* MA7327; ACO31296), PtnU4 (*S. platensis* MA7339; ADD83020), YtkF (*Streptomyces sp.* TP-A2060; ADZ13538), PdtorfI (*Pseudomonas stutzeri* KC; AAD39228), PdtI (*Pseudomonas putida*; AAQ01712), QbsK (*Pseudomonas fluorescens*; AAL65280), DddD (*Marinomonas MWYL1*; ZP_01598691), BbsE (*Thauera aromatica*; AAF89840), BbsF (*T. aromatica*; AAF89841), SmtA (*Chloroflexus aurantiacus*; ABF14399), SmtB (*C. aurantiacus*; ABF14400), Sct (*Chloroflexus aurantiacus* J-10-fl; ABY35475), CaiB-1 (*Archaeoglobus fulgidus* DSM 4304; AAB91206), CaiB (*E. coli*; CAA52112), BaiF (*Clostridium. scindens*; AAC45415), Mct (*C. aurantiacus* J-10-fl; A9WC36), MCR (*M. tuberculosis*; 1x74_A), HadA (*Clostridioides difficile* ATCC 9689; AAV40822), FldA (*Clostridium sporogenes*; AAL18808), Frc (*Oxalobacter formigenes*; AAC45298), YfdE (*E. coli*; 4HL6|A), YfdW (*E. coli* str. K-12 substr. MG1655; AAC75433), UctB (*Acetobacter aceti*; ABG35150), Act (*Variovorax paradoxus*; ACC69030). The sequence alignment was generated using ClustalW and the tree was generated using the Maximum Likelihood method based on the JTT Matrix-based method with 1000 bootstrap replications in MEGA. 7[4].

Enzymatic synthesis of aryl-CoA derivatives.

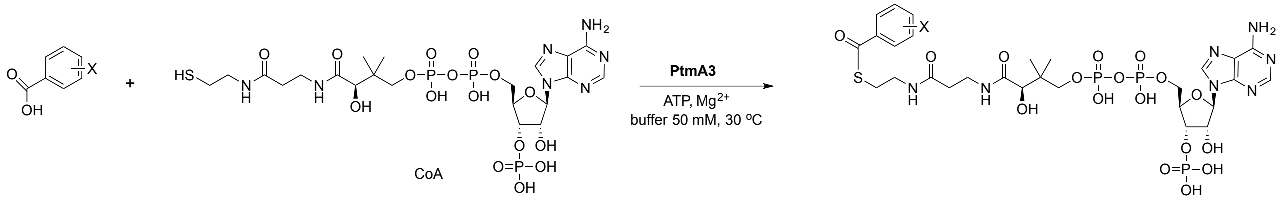

***S*-(3-Amino-2,4-dihydroxybenzoate) coenzyme A (5-CoA).** 3-Amino-2,4-dihydroxybenzoic acid (**5**, 10.0 mg, 0.059 mmol) was incubated with 20 μM PtmA3 in 10 mL of 50 mM phosphate, pH 7.6, containing 29.5 mg (0.058 mmol) ATP, 45.6 mg (0.058 mmol) CoA and 11.8 mg (0.058 mmol) $MgCl_2 \cdot 6H_2O$. After incubation at 30 °C for 2 h, the reaction was quenched with 10 mL of methanol and centrifuged. The supernatant was evaporated to 10 mL in vacuo at RT and purified by preparative HPLC using a 30 min gradient elution system of 0–10% CH_3CN in H_2O containing 10 mM NH_4OAc at a flow rate of 17 mL min^{-1} to afford 28.6 mg of **5-CoA** (t_R, 29.7 min, 52.8%) as a white, amorphous powder: $[\alpha]_D^{26}$ –8.4 (c 0.34, H_2O); UV (H_2O) λ_{max} (log ε) 324

(4.82), 257 (5.09) nm; IR (film) ν_{max} 3149, 1635, 1423, 1225, 1074, 947, 796, 720 cm^{-1}; ^{1}H NMR (700 MHz, D_2O): δ_H 8.42 (1H, s), 8.08 (1H, d, J = 0.7 Hz), 7.10 (1H, dd, J = 9.1, 1.4 Hz), 6.36 (1H, dd, J = 8.4, 1.4 Hz), 6.07 (1H, br d, J = 6.3 Hz), 4.83 (1H, m), 4.76 (1H, t, J = 6.3 Hz), 4.58 (1H, br s), 4.32 (2H, m), 4.00 (1H, br s), 3.87 (1H, dd, J = 9.8, 4.9 Hz), 3.60 (1H, dd, J = 9.8, 4.2 Hz), 3.50-3.44 (4H, m), 3.14 (2H, m), 2.47 (2H, t, J = 6.3 Hz), 0.93 (3H, s), 0.78 (3H, s) ppm; ^{13}C NMR (175 MHz, D_2O): δ_C 196.0, 174.7, 174.0, 155.2, 152.5, 151.6, 148.8, 148.5, 139.5, 130.9, 120.9, 118.3, 112.7, 107.7, 86.8, 83.6 (m), 74.3 (d, J = 6.6 Hz), 74.2, 73.1 (m), 71.9 (d, J = 6.3 Hz), 65.5 (d, J = 4.7 Hz), 38.7, 38.3 (d, J = 9.8 Hz), 35.3, 35.2, 27.6, 20.8, 18.2 ppm; HRESIMS affording the $[M + H]^+$ ion at *m/z* 919.1499 (calcd $[M + H]^+$ ion for $C_{28}H_{42}N_8O_{19}P_3S$ at *m/z* 919.1494).

***S*-(3-Amino-4-hydroxybenzoate) coenzyme A** (**6-CoA**). 3-Amino-4-hydroxybenzoic acid (**6**, 3.9 mg, 0.026 mmol) was incubated with 30 μM PtmA3 in 5 mL of 50 mM Tris, pH 8.0, containing 7.8 mg (0.015 mmol) ATP, 10.0 mg (0.013 mmol) CoA and 12.9 mg (0.064 mmol) $MgCl_2 \cdot 6H_2O$. After incubation at 30 °C for 3.5 h, the reaction was quenched with 5 mL of methanol and centrifuged. The supernatant was evaporated to 5 mL in vacuo at RT and purified by preparative HPLC using a 35 min gradient elution system of 2.5–18% CH_3CN in H_2O containing 10 mM NH_4OAc at a flow rate of 17 mL min^{-1} to afford 8.0 mg of **6-CoA** (t_R, 17.8 min, 69.8%) as a white, amorphous powder: $[\alpha]_D^{26}$ –7.8 (c 0.22, H_2O); UV (H_2O) λ_{max} (log ε) 324 (4.28), 247 (4.84) nm; IR (film) ν_{max} 3184, 1646, 1423, 1294, 1224, 1074, 947, 796 cm^{-1}; ^{1}H NMR (700 MHz, D_2O): δ_H 8.45 (1H, s), 8.13 (1H, br s), 7.24 (1H, m), 7.22 (1H, dt, J = 8.4, 2.1 Hz), 6.77 (1H, dd, J = 8.4, 1.4Hz), 6.08 (1H, dd, J = 6.3, 1.4 Hz), 4.82 (1H, m), 4.78 (1H, m), 4.58 (1H, s), 4.30 (2H, m), 4.00 (1H, br s), 3.85 (1H, dd, J = 9.8, 4.2 Hz), 3.58 (1H, dd, J = 9.8, 4.2 Hz), 3.49-3.43 (4H, m), 3.14 (2H, m), 2.46 (2H, t, J = 6.3 Hz), 0.91 (3H, s), 0.77 (3H, s) ppm; ^{13}C NMR (175 MHz, D_2O): δ_C 193.6, 174.7, 174.0, 155.3, 152.6, 150.7, 149.0, 139.5, 134.4, 128.4, 120.6, 118.5, 114.9, 114.5, 86.6, 83.7 (dd, J = 9.8, 3.3 Hz), 74.3 (d, J = 2.6 Hz), 74.2, 73.3 (d, J = 4.0 Hz), 71.9 (d, J = 5.8 Hz), 65.6 (d, J = 4.9 Hz), 38.8, 38.3 (d, J = 8.4 Hz), 35.4, 35.3, 28.0, 20.8, 18.2 ppm; HRESIMS affording the $[M + H]^+$ ion at *m/z* 903.1548 (calcd $[M + H]^+$ ion for $C_{28}H_{42}N_8O_{18}P_3S$ at *m/z* 903.1545).

***S*-(3-Amino-4-chlorobenzoate) coenzyme A (7-CoA).** 3-Amino-4-chlorobenzoic acid (**7**, 4.4 mg, 0.026 mmol) was incubated with 30 of μM PtmA3 in 5 mL of 50 mM Tris, pH 8.0, containing 7.8 mg (0.015 mmol) ATP, 10.0 mg (0.013 mmol) CoA and 12.9 mg (0.064 mmol) $MgCl_2 \cdot 6H_2O$. After incubation at 30 °C for 3 h, the reaction was quenched by boiling for 2 min and centrifuged. The supernatant was purified by preparative HPLC using a 35 min gradient elution system of 5–30% CH_3CN in H_2O containing 10 mM NH_4OAc at a flow rate of 17 mL min^{-1} to afford 9.2 mg of **7-CoA** (t_R, 18.5 min, 76.9%) as a white, amorphous powder: $[\alpha]_D^{26}$ –12.6 (c 0.25, H_2O); UV (H_2O) λ_{max} (log ε) 324 (4.09), 261 (5.06), 234 (5.09) nm; IR (film) ν_{max} 3184, 1646, 1427, 1227, 1047, 948, 796, 721 cm^{-1}; ^1H NMR (700 MHz, D_2O): δ_H 8.44 (1H, s), 8.12 (1H, d, J = 1.4 Hz), 7.26 (1H, dd, J = 8.4, 1.4 Hz), 7.24 (1H, br s), 7.12 (1H, dm, J = 8.4 Hz), 6.08 (1H, dd, J = 6.3, 1.4 Hz), 4.83 (1H, m), 4.80 (1H, m), 4.59 (1H, s), 4.30 (2H, m), 4.04 (1H, s), 3.87 (1H, dd, J = 9.8, 4.9 Hz), 3.61 (1H, dd, J = 9.8, 4.2 Hz), 3.51-3.44 (4H, m), 3.17 (2H, m), 2.47 (2H, t, J = 6.3 Hz), 0.94 (3H, s), 0.81 (3H, s) ppm; ^{13}C NMR (175 MHz, D_2O): δ_C 193.9, 174.7, 174.1, 155.1, 152.4, 148.9, 143.2, 139.5, 135.3, 129.5, 125.3, 118.4, 117.8, 114.3, 86.5, 83.4 (dd, J = 8.0, 3.2 Hz), 74.3, 74.0 (d, J = 4.6 Hz), 73.8 (dd, J = 7.5, 4.0 Hz), 71.8 (d, J = 6.1 Hz), 65.4 (d, J = 5.3 Hz), 38.6, 38.4 (d, J = 7.9 Hz), 35.4, 35.3, 28.3, 20.8, 18.4 ppm; HRESIMS affording the $[M - H]^-$ ion at *m/z* 919.1061 (calcd $[M - H]^-$ ion for $C_{28}H_{39}ClN_8O_{17}P_3S$ at *m/z* 919.1057).

***S*-(2-Amino-3-hydroxybenzoate) coenzyme A (8-CoA).** 2-Amino-3-hydroxybenzoic acid (**8**, 3.9 mg, 0.026 mmol) was incubated with 30 μM PtmA3 in 5 mL of 50 mM Tris, pH 8.0, containing 7.8 mg (0.015 mmol) ATP, 10.0 mg (0.013 mmol) CoA and 12.9 mg (0.064 mmol) $MgCl_2 \cdot 6H_2O$. After incubation at 30 °C for 3 h, the reaction was quenched with 5 mL of methanol and centrifuged. The supernatant was evaporated to 5 mL in vacuo at RT and purified by preparative HPLC using a 35 min gradient elution system of 2.5–30% CH_3CN in H_2O containing 10 mM NH_4OAc at a flow rate of 17 mL min^{-1} to afford 8.5 mg of **8-CoA** (t_R, 18.2 min, 74.2%) as a light yellow, amorphous powder: $[\alpha]_D^{26}$ –6.6 (c 0.39, H_2O); UV (H_2O) λ_{max} (log ε) 368 (4.25), 261 (4.87) nm; IR (film) ν_{max} 3184, 1643, 1548, 1423, 1211, 1074, 948, 851, 781, 725 cm^{-1}; ^1H NMR (700 MHz, D_2O): δ_H 8.46 (1H, s), 8.11 (1H, s), 7.31 (1H, dd, J = 8.4, 1.4 Hz), 6.91 (1H, dd, J = 7.1, 1.4 Hz), 6.55 (1H, t, J = 8.4 Hz), 6.09 (1H, d, J = 5.6 Hz), 4.84 (1H, m),

4.80 (1H, m), 4.59 (1H, s), 4.29 (2H, m), 4.01 (1H, s), 3.85 (1H, dd, J = 9.8, 4.9 Hz), 3.58 (1H, dd, J = 9.8, 4.9 Hz), 3.47 (2H, t, J = 6.3 Hz), 3.43 (2H, m), 3.11 (2H, m), 2.47 (2H, t, J = 6.3 Hz), 0.91 (3H, s), 0.78 (3H, s) ppm; ^{13}C NMR (175 MHz, D_2O): δ_C 193.9, 174.7, 174.0, 155.2, 152.4, 148.9, 144.0, 139.6, 137.5, 121.1, 118.8, 118.5, 118.4, 116.6, 86.6, 83.4 (dd, J = 8.4, 3.7 Hz), 74.2, 74.0 (d, J = 4.9 Hz), 73.7 (d, J = 3.3 Hz), 71.9 (d, J = 6.0 Hz), 65.3 (dd, J = 5.3 Hz), 38.8, 38.3 (dd, J = 8.8 Hz), 35.4, 35.3, 27.9, 20.8, 18.2 ppm; HRESIMS affording the $[M – H]^–$ ion at *m/z* 901.1400 (calcd $[M – H]^–$ ion for $C_{28}H_{40}N_8O_{18}P_3S$ at *m/z* 901.1405).

***S*-(2-Amino-4-fluorobenzoate) coenzyme A (9-CoA).** 2-Amino-4-fluorobenzoic acid (**9**, 4.0 mg, 0.026 mmol) was incubated with 30 µM PtmA3 in 5 mL 50 mM Tris, pH 8.0, containing 7.8 mg (0.015 mmol) ATP, 10.0 mg (0.013 mmol) CoA and 12.9 mg (0.064 mmol) $MgCl_2 \cdot 6H_2O$. After incubation at 30 °C for 3 h, the reaction was quenched by boiling for 2 min and centrifuged. The supernatant was purified by preparative HPLC using a 35 min gradient elution system of 5–30% CH_3CN in H_2O containing 10 mM NH_4OAc at a flow rate of 17 mL min^{-1} to afford 8.7 mg of **9-CoA** (t_R, 26.0 min, 74.0%) as a white, amorphous powder: $[\alpha]_D^{26}$ –8.9 (c 0.32, H_2O); UV (H_2O) λ_{max} (log ε) 348 (4.41), 261 (4.92) nm; IR (film) ν_{max} 3199, 1642, 1562, 1436, 1207, 1123, 1075, 984, 950, 880, 796, 723 cm^{-1}; ^{1}H NMR (700 MHz, D_2O): δ_H 8.46 (1H, s), 8.12 (1H, d, J = 1.4 Hz), 7.72 (1H, ddd, J = 9.1, 7.0, 1.4 Hz), 6.41 (1H, ddd, J = 11.2, 2.8, 1.4 Hz), 6.36 (1H, tdd, J = 8.4, 2.1, 1.4 Hz), 6.09 (1H, dd, J = 6.3, 1.4 Hz), 4.85 (1H, m), 4.80 (1H, m), 4.59 (1H, s), 4.30 (2H, m), 4.04 (1H, s), 3.88 (1H, dd, J = 9.8, 4.2 Hz), 3.61 (1H, dd, J = 9.8, 4.2 Hz), 3.47 (2H, t, J = 6.3 Hz), 3.42 (2H, t, J = 6.3 Hz), 3.10 (2H, m), 2.47 (2H, t, J = 6.3 Hz), 0.94 (3H, s), 0.81 (3H, s) ppm; ^{13}C NMR (175 MHz, D_2O): δ_C 192.6, 174.7, 174.0, 166.4 (d, J = 249 Hz), 155.2, 152.4, 150.5 (d, J = 13.3 Hz), 148.9, 139.6, 132.9 (d, J = 12.3 Hz), 118.5, 114.8, 104.7 (d, J = 23.6 Hz), 102.6 (d, J = 24.2 Hz), 86.5, 83.3 (dd, J = 8.4, 3.3 Hz), 74.2, 74.0 (d, J = 4.9 Hz), 73.7 (d, J = 2.8 Hz), 71.9 (d, J = 5.4 Hz), 65.3 (d, J = 4.9 Hz), 38.9, 38.3 (d, J = 7.7 Hz), 35.4, 35.3, 27.8, 20.8, 18.4 ppm; HRESIMS affording the $[M – H]^–$ ion at *m/z* 903.1356 (calcd $[M – H]^–$ ion for $C_{28}H_{39}FN_8O_{17}P_3S$ at *m/z* 903.1359).

***S*-(6-Hydroxy-2-naphthalenecarboxylate) coenzyme A (10-CoA).** 6-Hydroxy-2-naphthalene carboxylic acid (**10**, 4.8 mg, 0.026 mmol) was incubated with 30 µM PtmA3 in 5 mL of 50 mM Tris, pH 8.0, containing 7.8 mg (0.015 mmol) ATP, 10.0 mg (0.013 mmol) CoA and 12.9 mg

(0.064 mmol) $MgCl_2 \cdot 6H_2O$. After incubation at 30 °C for 3 h, the reaction was quenched by boiling for 2 min and centrifuged. The supernatant was purified by preparative HPLC using a 35 min gradient elution system of 5–30% CH_3CN in H_2O containing 10 mM NH_4OAc at a flow rate of 17 mL min^{-1} to afford 12.2 mg of **10-CoA** (t_R, 21.8 min, 100%) as a white, amorphous powder: $[\alpha]_D^{26}$ −11.2 (c 0.23, H_2O); UV (H_2O) λ_{max} (log ε) 324 (4.90), 261 (5.34) nm; IR (film) ν_{max} 3196, 1644, 1475, 1435, 1224, 1117, 1072, 946, 887, 722 cm^{-1}; ^1H NMR (700 MHz, D_2O): δ_H 8.25 (1H, s), 8.05 (1H, s), 7.82 (1H, s), 7.65 (1H, d, J = 9.1 Hz), 7.56 (1H, dd, J = 8.4, 2.1 Hz), 7.46 (1H, d, J = 8.4 Hz), 7.08 (1H, dd, J = 8.4, 2.1 Hz), 7.04 (1H, br s), 5.86 (1H, d, J = 6.3 Hz), 4.81 (1H, m), 4.69 (1H, t, J = 6.3 Hz), 4.55 (1H, s), 4.30 (2H, m), 4.03 (1H, s), 3.87 (1H, dd, J = 9.8, 4.9 Hz), 3.61 (1H, dd, J = 9.8, 4.2 Hz), 3.51-3.45 (4H, m), 3.21 (2H, m), 2.49 (2H, t, J = 6.3 Hz), 0.93 (3H, s), 0.80 (3H, s) ppm; ^{13}C NMR (175 MHz, D_2O): δ_C 194.1, 174.7, 174.4, 156.0, 154.3, 151.5, 148.2, 139.2, 137.2, 131.6, 130.7, 128.4, 126.6, 126.6, 122.8, 118.9, 118.0, 109.1, 86.5, 83.1 (dd, J = 9.3, 3.3 Hz), 74.2, 74.0 (d, J = 4.7 Hz), 73.7 (m), 71.9 (d, J = 5.8 Hz), 65.2 (d, J = 5.1 Hz), 38.8, 38.4 (d, J = 8.6 Hz), 35.4, 35.3, 28.2, 20.8, 18.4 ppm; HRESIMS affording the $[M - H]^-$ ion at m/z 936.1447 (calcd $[M - H]^-$ ion for $C_{32}H_{41}N_7O_{18}P_3S$ at m/z 936.1454).

Enzymatic synthesis of 3-amino-2,4-dihydroxythiobenzoic acid.

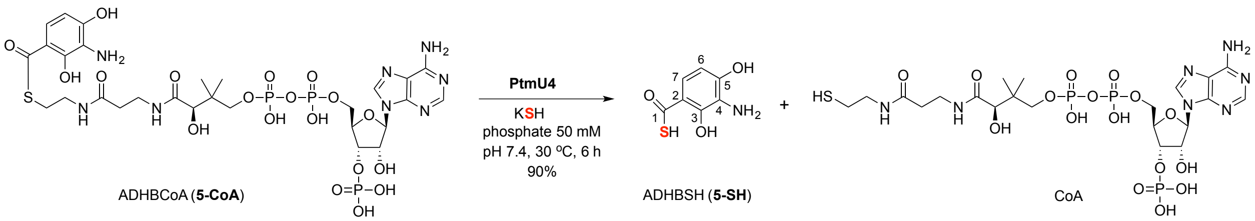

3-Amino-2,4-dihydroxythiobenzoic acid (5-SH). *S*-(3-Amino-2,4-dihydroxybenzoate) coenzyme A (**5-CoA**, 11 mg, 0.012 mmol) was incubated with 10 µM PtmU4 in 5 mL of 50 mM phosphate, pH 7.4, containing 8.6 mg (0.12 mmol) KSH. After incubation at 30 °C for 6 h, the reaction was quenched by boiling for 2 min and centrifuged. The supernatant was purified by preparative HPLC using a 40 min gradient elution system of 0–10% CH_3CN in H_2O containing 10 mM NH_4OAc at a flow rate of 10 mL min^{-1} to afford 2.0 mg of **5-SH** (t_R, 11.9 min, 90%) as a light yellow, amorphous powder: UV (H_2O) λ_{max} (log ε) 309 (5.14), 242 (5.19), 219 (5.25) nm; IR (film) ν_{max} 3184, 1575, 1458, 1330, 1260, 1234, 1193, 1041, 843, 800, 759, 723 cm^{-1}; ^1H NMR (700 MHz, D_2O): δ_H 7.87 (1H, d, J = 9.1 Hz, H-7), 6.52 (1H, d, J = 9.1 Hz, H-6) ppm; ^{13}C

NMR (175 MHz, D_2O) δ_C 212.6 (s, C-1), 151.6 (s, C-5), 151.3 (s, C-3), 127.4 (d, C-7), 114.4 (s, C-4), 118.6 (s, C-2), 106.2 (d, C-6) ppm; HRESIMS affording the $[M + H]^+$ ion at *m/z* 186.0223 (calcd $[M + H]^+$ ion for $C_7H_8NO_3S$ at *m/z* 186.0219).

Isolation and structural elucidation of thioPTM (1) and thioPTN (2). ThioPTM (**1**) was isolated from a large-scale fermentation (0.8 L) of the *ΔptmS2* mutant, SB12042. Two 2.0 L baffled flasks, each containing 400 mL of production medium with 4% (v/v) seed culture and 15 g of Amberlite XAD-16 resin, were incubated for 2.5 days. The harvested and washed resin was extracted three times with ca. 150 mL of CH_3OH. Methanol was removed in vacuo at room temperature and the resulting oil was stored at –80 °C freezer until further purification. A quarter of the oil was dissolved in DMSO–CH_3OH (1:1). The precipitate was removed and the supernatant was fractionated by preparative reverse-phase HPLC using a 30 min elution system of 10–70% CH_3CN in H_2O at a flow rate of 17 mL min^{-1}. The fractions containing thioPTM were pooled and the solvent was removed in vacuo at room temperature. Then another preparative reverse-phase HPLC was performed using a 30 min elution system of 0–60% CH_3OH in H_2O, in which the thioPTM was eluted at t_R = 25 min. A white and amorphous solid of thioPTM (10.0 mg) was obtained after removing the water using a lyophilizer.

ThioPTN (**2**) was isolated from a large-scale fermentation (0.5 L) of the PTM-PTN dual overproducer *S. platensis* SB12026[5]. *S. platensis* SB12026 was cultured in seed medium [20 g dextrose, 15 g yeast extract (Difco), 10 g malt extract, 0.244 g $MgSO_4 \cdot 7H_2O$ and 0.3 g $FeCl_3 \cdot 6H_2O$ in 1 L deionized water, pH 7.0] for 2 days at 30 °C (50 mL in 250 mL flasks), and then 2 mL of the resulting culture was inoculated to 50 mL production medium [20 g soluble starch, 10 g corn steep liquor (Sigma), 0.5 g KH_2PO_4 and 0.25 g $MgSO_4$, supplemented with 1 mL trace element solution in 1 L deionized water, pH 7.0]. After 7 days fermentation, the resins were filtered out from the fermentation broth using paper towel, and immediately transferred to –80 °C refrigerator. After 2 hours, the frozen resins were dried in a lyophilizer for 2 days. The dried resins (14.0 g) were then added to a 50 mL round-bottom flask and washed with acetone (30 mL) for five times. The filtrates were combined and dried with sodium sulfate and concentrated under vacuum. Then the residue was re-dissolved in 30 mL dichloromethane (DCM) and filtered again. The filtrates was concentrated under vacuum to generate the crude

extracts, which was dissolved in 4 mL of DCM and fractionated by silica gel column chromatography [2×30 cm, eluted with DCM : trimethylamine (TEA) = 25 : 1]. A partial pure thioPTN (40 mg) was eluted from the first pale yellow fraction. A portion (10.0 mg) of this fraction was purified by preparative reverse-phase HPLC using a 35 min elution system of 30–85% CH_3OH in H_2O at a flow rate of 17 mL min^{-1}, in which the thioPTN was eluted at $t_R = 28$ min. A white and amorphous solid of thioPTN (5.0 mg) was obtained after removing the water using a lyophilizer.

Thioplatensimycin (1). White, amorphous solid; $[\alpha]_D^{26}$ −17.4 (c 0.2, CH_3OH); UV (CH_3OH) λ_{max} (log ε) 318 (4.69), 240 (4.96) nm; IR (film) ν_{max} 3289, 2963, 1652, 1532, 1447, 1379, 1244, 1078, 1044, 953, 830, 678 cm^{-1}; ^1H and ^{13}C NMR data, see Supplementary Table 4; HRESIMS affording the $[M + H]^+$ ion at *m/z* 458.1634 (calcd $[M + H]^+$ for $C_{24}H_{28}NO_6S$ at 458.1634).

Thioplatensin (2). White, amorphous solid; $[\alpha]_D^{26}$ −5.8 (c 0.08, DMSO); UV (CH_2Cl_2) λ_{max} (log ε) 316 (4.92), 242 (5.11) nm; IR (film) ν_{max} 2931, 1655, 1532, 1377, 1232, 1080, 827 cm^{-1}; ^1H and ^{13}C NMR data, see Supplementary Table 5; HRESIMS affording the $[M + H]^+$ ion at *m/z* 442.1681 (calcd $[M + H]^+$ for $C_{24}H_{28}NO_5S$ at 442.1683).

Supplementary Tables

Supplementary Table 1. Strains used in this study.

Strain	Genotype, Description	Source (Reference)
E. coli DH5α	*E. coli* host for general cloning	Life Technologies
E. coli BL21 (DE3)	*E. coli* host for protein expression	Life Technologies
E. coli BW25113/pIJ790	*E. coli* host for λRED-mediated PCR targeting	(6)
E. coli ET12567/pUZ8002	Methylation-deficient *E. coli* host for intergeneric conjugation	(7)
S. platensis SB12026	CB00739 Δ*ptmR1::aac(3)IV*	(5)
S. platensis SB12029	CB00739 Δ*ptmR1* (markerless)	(8)
S. platensis SB12039	SB12029 Δ*ptmA3* (markerless)	This study
S. platensis SB12040	SB12029 Δ*ptmU4::aac(3)IV*	This study
S. platensis SB12041	SB12029 Δ*ptmS1::aac(3)IV*	This study
S. platensis SB12042	SB12029 Δ*ptmS2::aac(3)IV*	This study
S. platensis SB12043	SB12029 Δ*ptmS4::aac(3)IV*	This study
S. platensis SB12044	SB12029 Δ*ptmS3::aac(3)IV* disruption mutant	This study
S. albus J1074	*Streptomyces* host for ADHBSH biotransformation	(9)
S. lividans K4-114	*Streptomyces* host for ADHBSH biotransformation	(10)
S. avermitilis SUKA22	*Streptomyces* host for ADHBSH biotransformation and protein expression	(11)
S. coelicolor M1146	*Streptomyces* host for ADHBSH biotransformation	(12)
S. albus SB12303	*S. albus* J1074 containing pBS12086 for ADHBSH biotransformation	This study
S. lividans SB12304	*S. lividans* K4-114 containing pBS12086 for ADHBSH biotransformation	This study
S. coelicolor SB12305	*S. coelicolor* M1146 containing pBS12086 for ADHBSH biotransformation	This study
S. avermitilis SB12306	*S. avermitilis* SUKA22 containing pBS12086 for ADHBSH biotransformation	This study
S. avermitilis SB12307	*S. avermitilis* SUKA22 containing pBS12092 for PtmU4 protein production	This study
S. aureus ATCC 25923	Methicillin sensitive strain for antibacterial assay	ATCC
K. rhizophila ATCC 9431	Strain for antibacterial assay	ATCC

Supplementary Table 2. Plasmids and cosmids used in this study.

Plasmid	Description	Source (Reference)
pIJ773	Plasmid containing the apramycin resistance cassette (*aac(3)IV+oriT*)	(6)
pOJ260	*E. coli*-*Streptomyces* shuttle vector	(13)
pUWL201PW	*E. coli*-*Streptomyces* expression shuttle vector	(14)
pUWL201PWT	*E. coli*-*Streptomyces* expression shuttle vector harboring *oriT* (cloned into the *Pst*I site)	(8)
pBS12037	Cosmid 18H9 containing a partial *ptm* gene cluster and Δ*ptmR1* (markerless)	(8)
pBS12074	pBS12037 Δ*ptmU4::aac(3)IV*	This study
pBS12075	pOJ260-derived for inactivation of *ptmA3*	This study
pBS12076	Cosmid 8E1 for inactivation of *ptmS4*	This study
pBS12077	Cosmid 16A4 for inactivation of *ptmS1* and *ptmS2*	This study
pBS12078	Cosmid 6C10 for inactivation of *ptmS3*	This study
pBS12079	pBS12079 Δ*ptmS4::aac(3)IV*	This study
pBS12080	pBS12080 Δ*ptmS1::aac(3)IV*	This study
pBS12081	pBS12080 Δ*ptmS2::aac(3)IV*	This study
pBS12082	pBS12081 Δ*ptmS3::aac(3)IV*	This study
pET-44b(+)	Plasmid for cloning and protein expression	Novagen
pBS12083	pET-44b(+) harboring *ptmU4*	This study
pBS12084	pET-44b(+) harboring *ptmU4+A3*	This study
pBS12085	pUWL201PWT harboring *ptmU4+A3*	This study
pBS12086	pUWL201PWT harboring *ptmU4+A3* and *ptmB1-B3*	This study
pBS3080	pRSFDuet-1 derived plasmid containing a *Bsm*FI site for ligation-independent cloning (LIC) and encodes a TEV protease site after the N-terminal His_6-tag	(15)
pBS12087	pBS3080 harboring the *ptmA3*; used for enzyme assays	This study
pBS12088	pBS3080 harboring *ptmU4*	This study
pBS12089	pBS3080 harboring *ptmS4*	This study
pBS12090	pBS3080 harboring the truncated *ptmS2* (residue 1–90)	This study
pBS12091	pBS3080 harboring the truncated *ptmS3* (residue 1–91)	This study
pBS12092	pUWL201PWT harboring full length of *ptmU4*; used for enzyme assays	This study
pBS12093	pBS12098-derived *ptmU4* D430A mutant	This study
pBS12094	pBS12098-derived *ptmU4* D430E mutant	This study
pBS12095	pBS12098-derived *ptmU4* D430N mutant	This study
pBS12096	pBS3080 harboring *fabF* C163Q mutant	This study

Supplementary Table 3. Protein BLAST (BLASTP) analysis of PtmS1–S4 homologues in the genome of *S. avermitilis*.[a]

Protein	Number of homologues	identity / similarity (%)	protein accession number
PtmS1 homologue	1	90 / 94	WP_010986566
PtmS2 homologues	2	82 / 91	WP_010986567
		58 / 74	WP_010985363
PtmS3 homologues	3	89 / 94	WP_010985363
		55 / 72	WP_010986567
		28 / 41	WP_010985469
PtmS4 homologue	1	93 / 96	WP_010984512

[a] The protein accession numbers of PtmS1–S4 are AVR47602-AVR47605, respectively. The genes encoding homologues of PtmS1 (WP_010986566) and PtmS2 (WP_010986567) clustered together in the genome of *S. avermitilis*.

Supplementary Table 4. ^{13}C NMR (175 MHz) and ^{1}H NMR (700 MHz) spectroscopic data for thioplatensimycin (**1**, thioPTM) and platensimycin (**3**, PTM) in CD_3OD (δ in ppm, J in Hz). [a]

	**1**		**3**	
No.	δ_C	δ_H	δ_C	δ_H
1	175.9, s		175.9, s	
2a	32.3, t	2.50, m[b]	32.2, t	2.49, m[b]
2b		2.33, m[b]		2.32, m[b]
3a	33.0, t	2.33, m[b]	32.9, t	2.32, m[b]
3b		1.90, m[b]		1.90, m[b]
4	48.0, s		48.0, s	
5	205.9, s		205.9, s	
6	128.0, d	5.90, d (10.5)	128.0, d	5.90, d (10.5)
7	156.2, d	6.65, d (11.2)	156.2, d	6.66, d (10.5)
8	47.5, s		47.5, s	
9	47.5, d	2.45, br s	47.5, d	2.45, br s
10	78.2, d	4.53, br s	78.2, d	4.53, br s
11a	41.6, t	2.12, m[b]	41.6, t	2.12, m[b]
11b		2.10, m[b]		2.10, m[b]
12	46.3, d	2.45, m[b]	46.3, d	2.45, m[b]
13a	44.1, t	2.08, br d (11.9)	44.1, t	2.08, br d (11.9)
13b		1.87, m		1.87, m
14a	56.0, t	1.83, br d (11.2)	56.0, t	1.83, br d (11.2)
14b		1.74, br d (11.2)		1.74, br d (11.2)
15	88.9, s		88.9, s	
16	23.3, q	1.44, s	23.3, q	1.44, s
17	25.3, q	1.27, s	25.3, q	1.27, s
1′	212.7, s		173.8, s	
2′	120.3, s		106.4, s	
3′	156.5, s		159.6, s	
4′	113.9, s		114.0, s	
5′	156.8, s		159.7, s	
6′	108.3, d	6.31, d (9.1)	109.6, d	6.44, d (9.1)
7′	132.6, d	8.11, d (9.1)	130.7, d	7.65, d (9.1)

[a] Assignments are based on 1D and 2D NMR experiments.
[b] Signals are overlapped.

Supplementary Table 5. ^{13}C NMR (175 MHz) and ^{1}H NMR (700 MHz) spectroscopic data for thioplatencin (**2**, thioPTN) and platencin (**4**, PTN) in CD_3OD (δ in ppm, J in Hz).[a]

	**2**		**4**	
No.	δ_C	δ_H	δ_C	δ_H
1	176.1, s		176.2, s	
2a	32.3, t	2.38, m[b]	32.2, t	2.37, m[b]
2b		2.35, m[b]		2.34, m[b]
3a	32.1, t	2.15, m[b]	32.0, t	2.16, m[b]
3b		1.74, m[b]		1.74, m[b]
4	48.9, s		49.1, s	
5	207.0, s		207.0, s	
6	127.0, d	5.86, d (9.8)	127.0, d	5.86, d (9.8)
7	157.2, d	6.64, d (9.8)	157.3, d	6.65, d (9.8)
8	37.6, s		37.6, s	
9	41.0, d	2.14, m[b]	41.0, d	2.14, m[b]
10a	27.7, t	2.03, m	27.7, t	2.03, m
10b		1.57, td (12.6, 3.5)		1.57, td (12.6, 3.5)
11	37.7, d	2.43, br s	37.7, d	2.44, br s
12a	27.1, t	1.82, m[b]	27.1, t	1.82, m[b]
12b		1.74, m		1.74, m
13a	29.1, t	1.82, m[b]	29.1, t	1.82, m[b]
13b		1.66, t (11.2)		1.66, t (11.2)
14a	45.6, t	2.39, br d (15.4)	45.7, t	2.40, br d (15.4)
14b		2.13, br d (15.4)		2.13, br d (15.4)
15	150.6, s		150.7, s	
16a	107.8, t	4.86, br s	107.8, t	4.86, br s
16b		4.69, br s		4.69, br s
17	22.1, q	1.21, s	22.1, q	1.21, s
1′	n.d.[c]		173.7, s	
2′	118.5, s		106.2, s	
3′	157.7, s		159.6, s	
4′	114.1, s		114.1, s	
5′	157.7, s		159.6, s	
6′	108.5, d	6.35, d (9.1)	109.7, d	6.43, d (9.1)
7′	131.8, d	8.02, d (9.1)	130.6, d	7.65, d (9.1)

[a] Assignments are based on 1D and 2D NMR experiments.
[b] Signals are overlapped.
[c] Signals are not detected.

Supplementary Figures

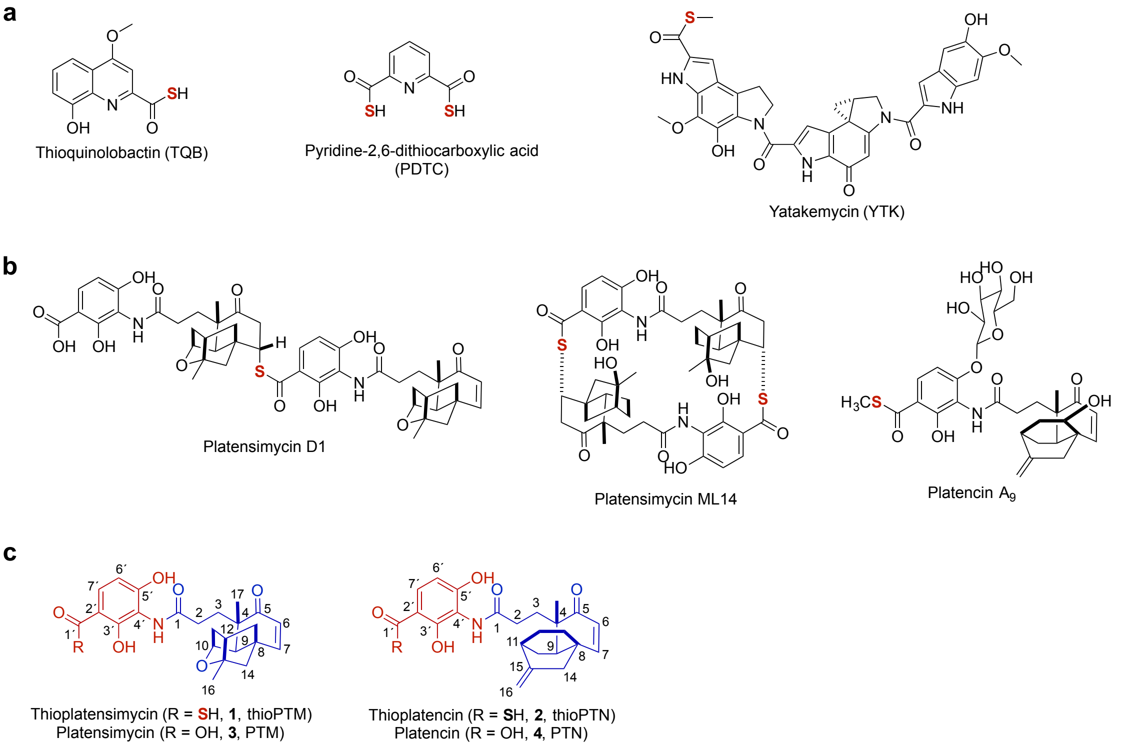

Supplementary Figure 1. Selected thiocarboxylic acid or thiocarboxylic acid-derived natural products. **a**, Selected thiocarboxylic acid-containing natural products. The biosynthetic gene clusters of TQB, PDTC, and YTK are *qbs*, *pdt*, and *ytk*, respectively. Yatakemycin is a methyl thioester derivative, the generation of which was proposed from methylation of a thiocarboxylic acid intermediate[16]. **b**, Structures of other thioPTM- and thioPTN-derived congeners isolated from the recombinant *S. platensis* strains. Platensimycin D1, a thioPTM pseudodimer, was isolated from SB12029[1]; platensimycin ML14, a cyclic thioPTM dimer, was isolated from SB12036[17]; platencin A_9, a thioPTN methyl thioester, was isolated from the PTN-exclusive overproducer *S. platensis* SB12600[18]. **c**, Carbon labeling of thioPTM (**1**), thioPTN (**2**), PTM (**3**), and PTN (**4**).

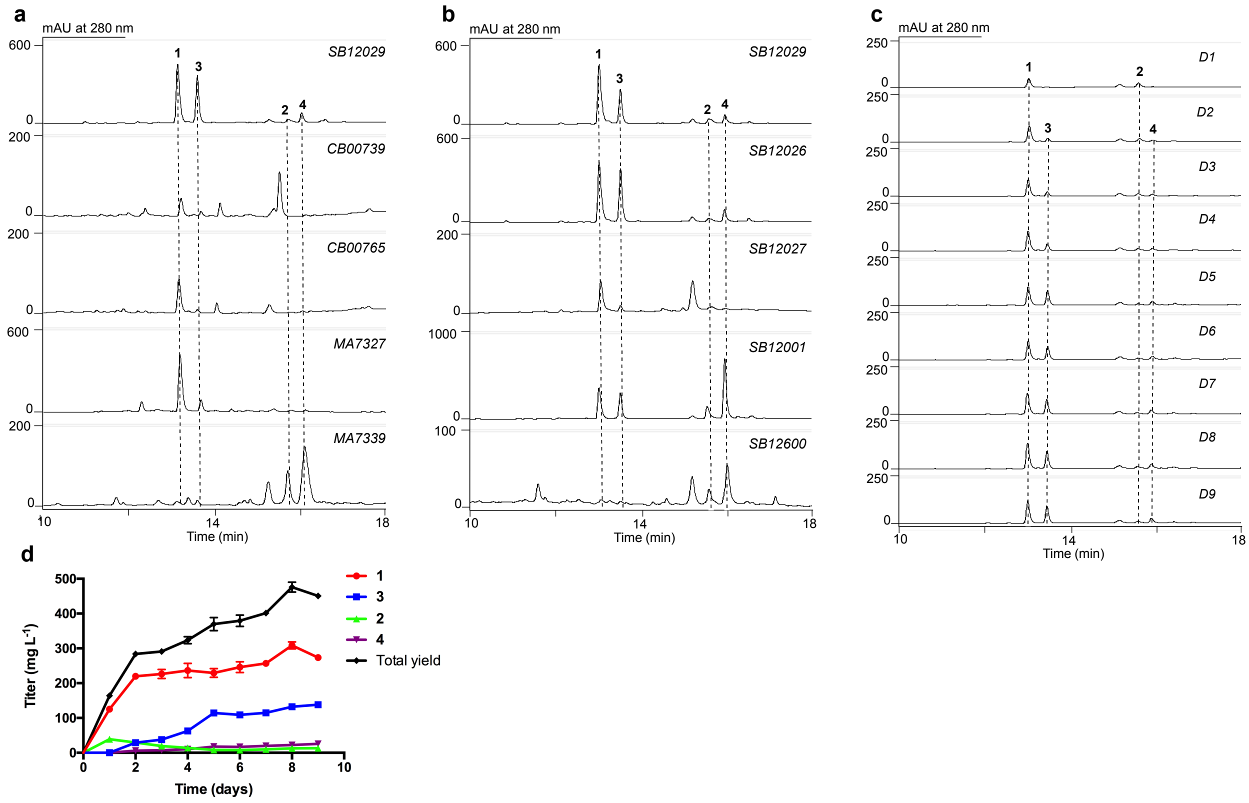

Supplementary Figure 2. Fermentation evidence of the genetically-encoded production of thioPTM and thioPTN. **a**, LC-MS analysis of crude extracts from the PTM–PTN dual overproducer *S. platensis* SB12029, which was previously constructed by in-frame deletion of the pathway-specific negative regulator *ptmR1* in *S. platensis* CB00739, as well as the original (*S. platensis* MA7327) and two alternate wild-type PTM-PTN dual (*S. platensis* CB00739 and CB00765) or PTN-exclusive producing *Streptomyces platensis* strains (*S. platensis* MA7339). **b**, LC-MS analysis of crude extracts from the PTM–PTN dual overproducers *S. platensis* SB12029, SB12026 (CB00739 Δ*ptmR1::aac(3)IV*), SB12027 (CB00765 Δ*ptmR1::aac(3)IV*), and SB12001 (MA7327 Δ*ptmR1::aac(3)IV*), and the PTN-exclusive overproducer *S. platensis* SB12600 (MA7339 Δ*ptmR1::aac(3)IV*). **c**, LC-MS analysis of crude extracts harvested from day 1 (D1) to day 9 (D9) of *S. platensis* SB12029. **d**, Titers of **1–4**, as well as the combined yield; the highest total yield appeared on day 8 with the production of 308 ± 10 mg L^{-1} of **1**, 132 ± 1 mg L^{-1} of **3**, 13 ± 3 mg L^{-1} of **2**, and 22 ± 1 mg L^{-1} of **4.** All experiments were carried out in duplicate with standard deviations listed.

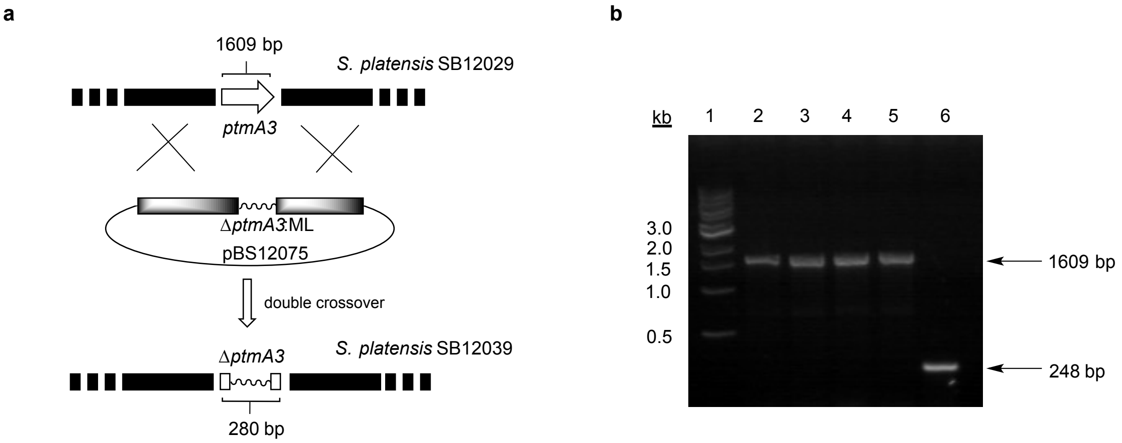

Supplementary Figure 3. In-frame inactivation of *ptmA3* in the PTM-PTN overproducer *S. platensis* SB12029 affording *S. platensis* SB12039. **a**, Schematic representation for the in-frame deletion of *ptmA3* in *S. platensis* SB12029; **b**, PCR verification of wild-type (1609 bp) and double crossover (280 bp) mutant genotypes using the primers 739A3ID_F and 739A3ID_R. Lane 1, 1 kb DNA ladder (NEB); lanes 2 to 5, *S. platensis* SB12029; lane 6, *S. platensis* SB12039.

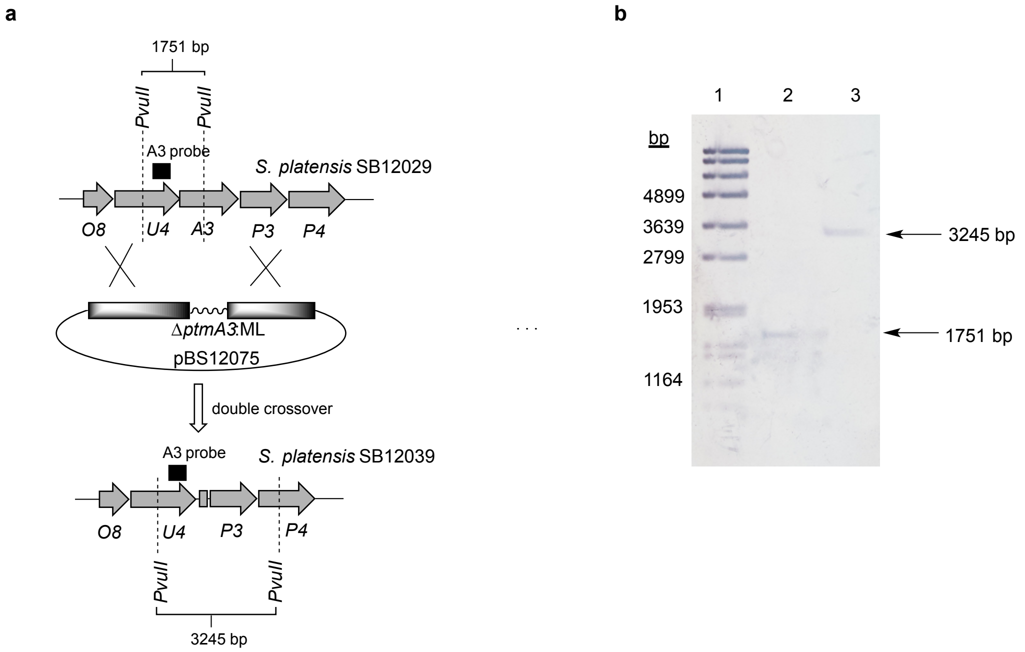

Supplementary Figure 4. Southern analysis of the Δ*ptmR1*/Δ*ptmA3* double mutant *S. platensis* SB12039. **a**, Schematic representation for the in-frame deletion of *ptmA3* in *S. platensis* SB12029. The probe for *ptmA3* (468 bp) was amplified using the primers 739A3south_F and 739A3south_R and genomic DNAs as the template; **b**, Southern blot verification of wild-type *ptmA3* (1751 bp) and double crossover Δ*ptmA3* (3245 bp) mutant genotypes. Lane 1, DNA marker VII, DIG-labeled (Roche); lane 2, *S. platensis* SB12029; lane 3, *S. platensis* SB12039.

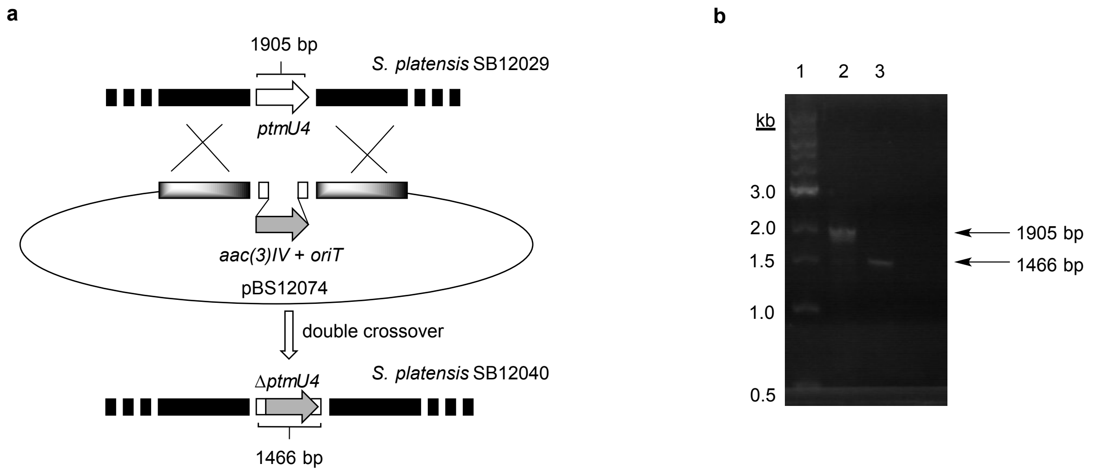

Supplementary Figure 5. Inactivation of *ptmU4* in the PTM-PTN overproducer *S. platensis* SB12029 affording *S. platensis* SB12040. **a**, Schematic representation for the deletion of *ptmU4* in *S. platensis* SB12029 by insertion of an apramycin resistance cassette [*aac(3)IV* + *oriT*]; **b**, PCR verification of wild-type (1905 bp) and double crossover (1466 bp) mutant genotypes using the primers 739U4ID_F and 739U4ID_R. Lane 1, 1 kb DNA ladder (NEB); lane 2, *S. platensis* SB12029; lane 3, *S. platensis* SB12040.

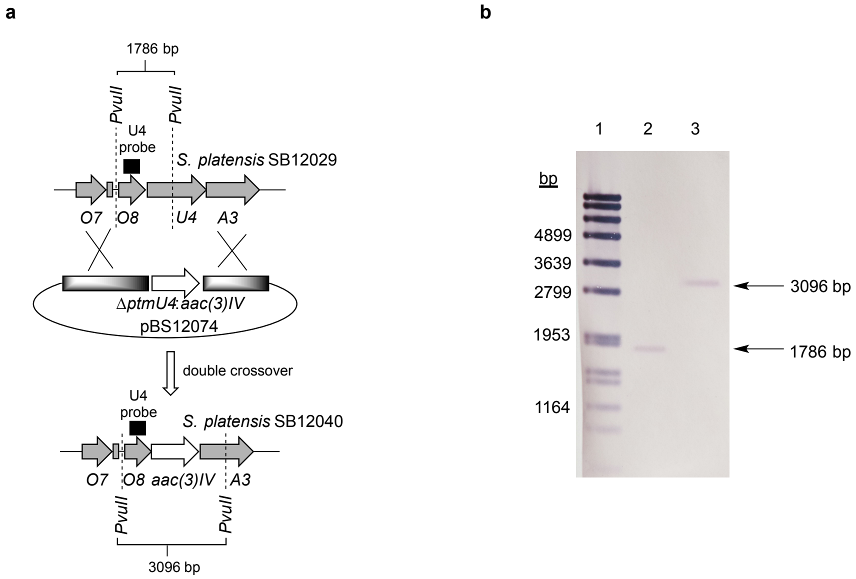

Supplementary Figure 6. Southern analysis of the Δ*ptmR1*/Δ*ptmU4* double mutant *S. platensis* SB12040. **a**, Schematic representation for the deletion of *ptmU4* in *S. platensis* SB12029 by insertion of an *aac(3)IV* + *oriT* cassette. The probe for *ptmU4* (470 bp) was amplified using the primers 739U4south_F and 739U4south_R and genomic DNAs as the template; **b**, Southern blot verification of wild-type *ptmU4* (1786 bp) and double crossover Δ*ptmU4* (3096 bp) mutant genotypes. Lane 1, DNA marker VII, DIG-labeled (Roche); lane 2, *S. platensis* SB12029; lane 3, *S. platensis* SB12040.

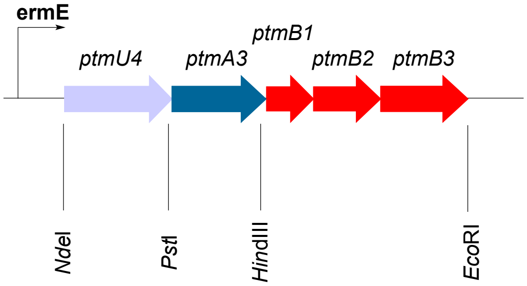

Supplementary Figure 7. Schematic representation of the design and construction of the ADHBSH (**5-SH**) production system in model *Streptomyces*.

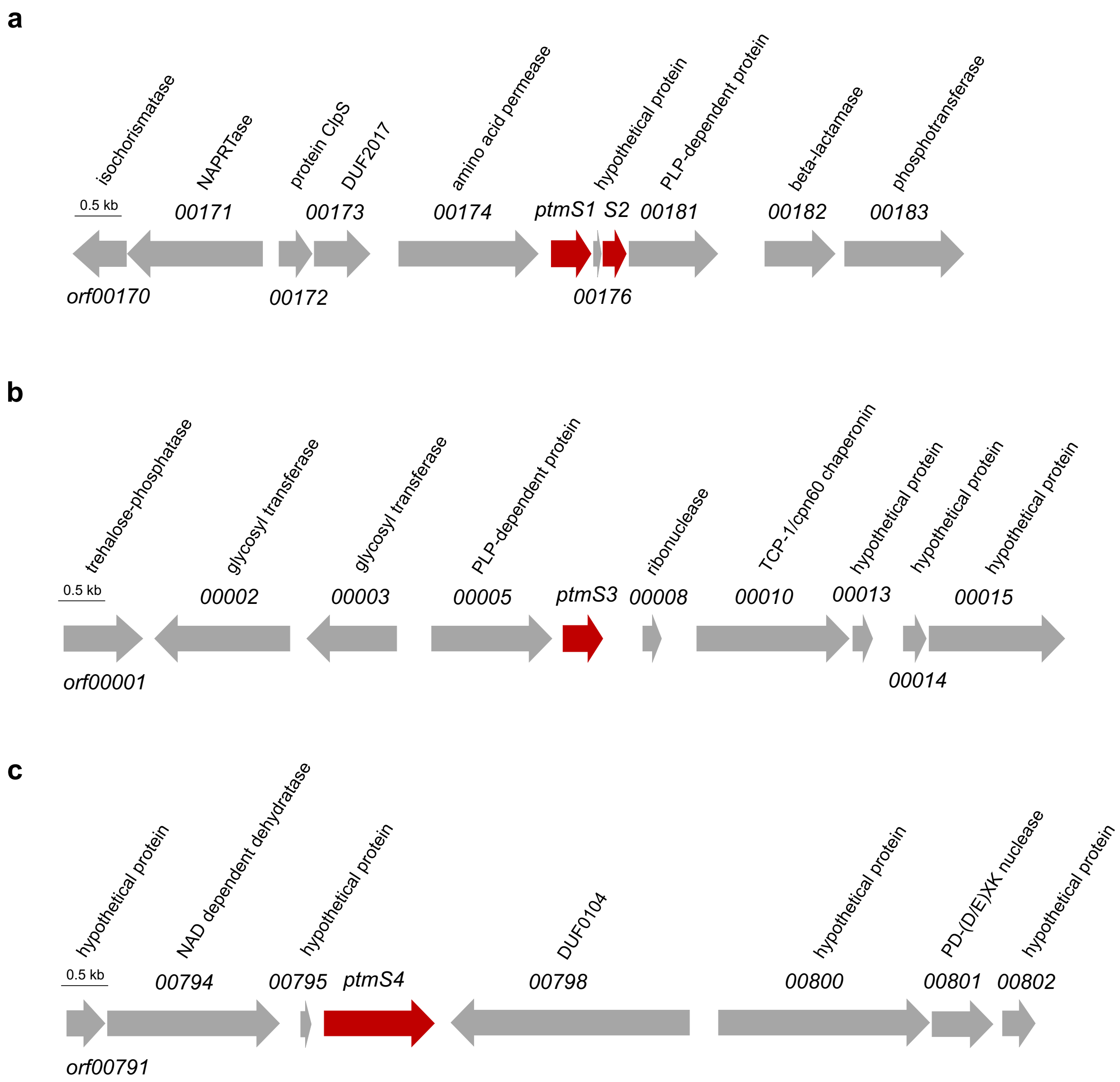

Supplementary Figure 8. Locations of *ptmS1–S4* in the genome of *S. platensis* CB00739. **a**, *ptmS1*, which encodes a JAMM family metalloprotease, clusters together with the capped sulfur-carrier protein encoding gene *ptmS2*. **b**, The location of *ptmS3* in in the genome of *S. platensis* CB00739. **c**, The location of *ptmS4* in in the genome of *S. platensis* CB00739.

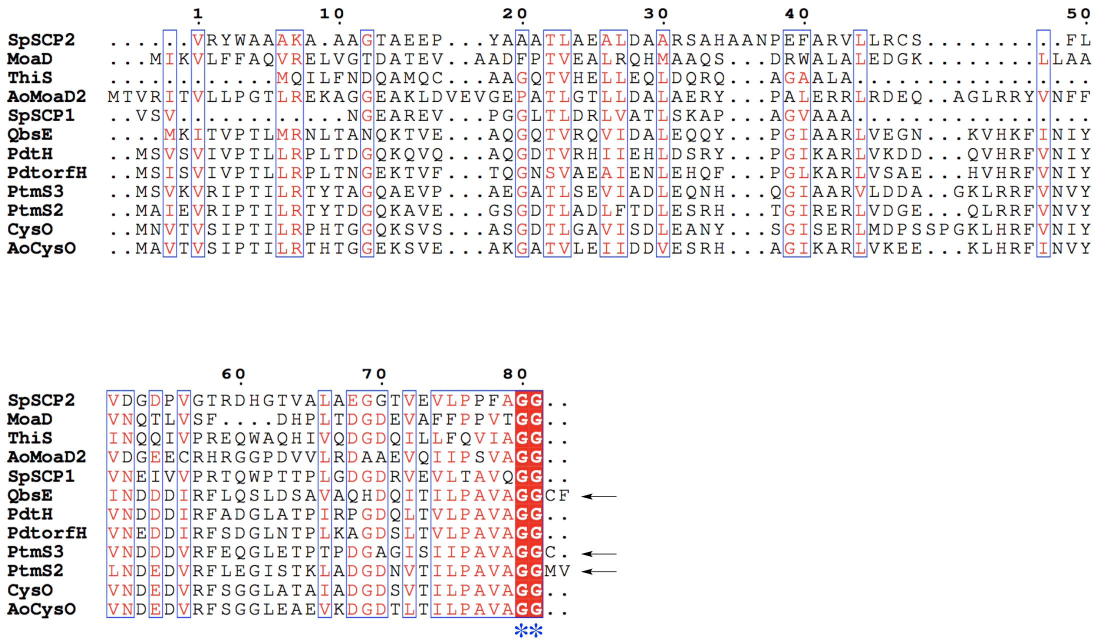

Supplementary Figure 9. Sequence alignment of selected sulfur-carrier proteins from bacteria. Aligned residues are colored based on the level of conservation (red box with white character shows strict identity, red character similarity, and blue frame similarity across groups). The conserved GG motif is shown with blue asterisks. The three capped sulfur-carrier proteins (PtmS2, PtmS3, and QbsE), which have additional amino acids at C-terminal to the required GG motif, are indicated with black arrows. The protein sequences were obtained from the NCBI database: PtmS2 (*S. platensis* CB00739; MG265943), PtmS3 (*S. platensis* CB00739; MG265944), SpSCP1 (*S. platensis* CB00739; MG265946), SpSCP2 (*S. platensis* CB00739; MG265947), QbsE (*P. fluorescens*; AAL65287), PdtorfH (*P. stutzeri* KC; AAD39227), PdtH (*P. putida*; AAQ01711), ThiS (*E. coli str.* K-12 *substr.* MG1655; YP_026279), MoaD (*E. coli str.* K-12 *substr.* MG1655; NP_415305), CysO (*M. tuberculosis* H37Rv; NP_215851), AoCysO (*Amycolatopsis orientalis subsp.* vinearia; AFO69367), and AoMoaD2 (*A. orientalis subsp.* vinearia; AFO69382). The alignment was created with MUSCLE[19] and rendered with ESPript 3.0[20].

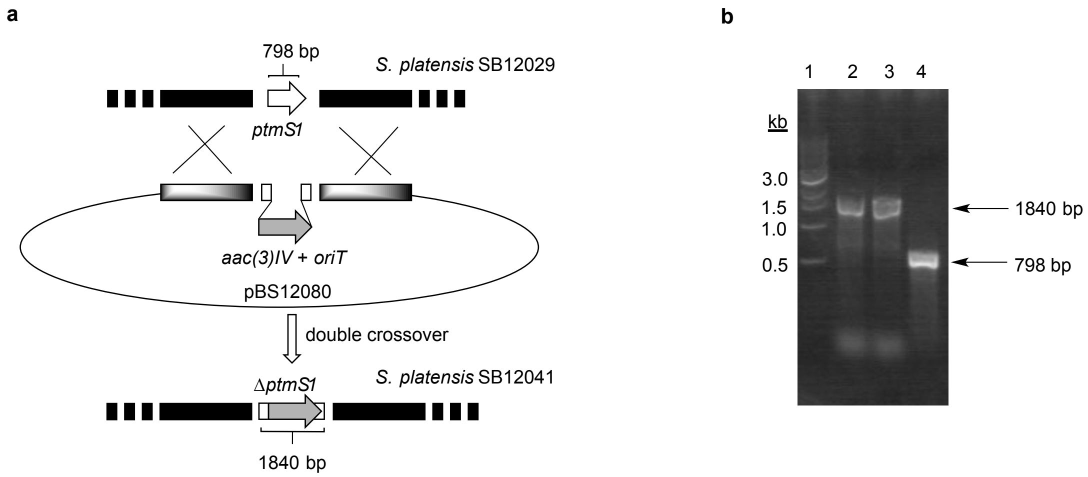

Supplementary Figure 10. Inactivation of *ptmS1* in the PTM-PTN overproducer *S. platensis* SB12029 affording *S. platensis* SB12041. **a**, Schematic representation for the deletion of *ptmS1* in *S. platensis* SB12029 by insertion of an apramycin resistance cassette [*aac(3)IV* + *oriT*]; **b**, PCR verification of wild-type (798 bp) and double crossover (1840 bp) mutant genotypes using the primers 739*ptmS1*ID_F and 739*ptmS1*ID_R. Lane 1, 1 kb DNA ladder (NEB); lanes 2 and 3, *S. platensis* SB12041; lane 4, *S. platensis* SB12029.

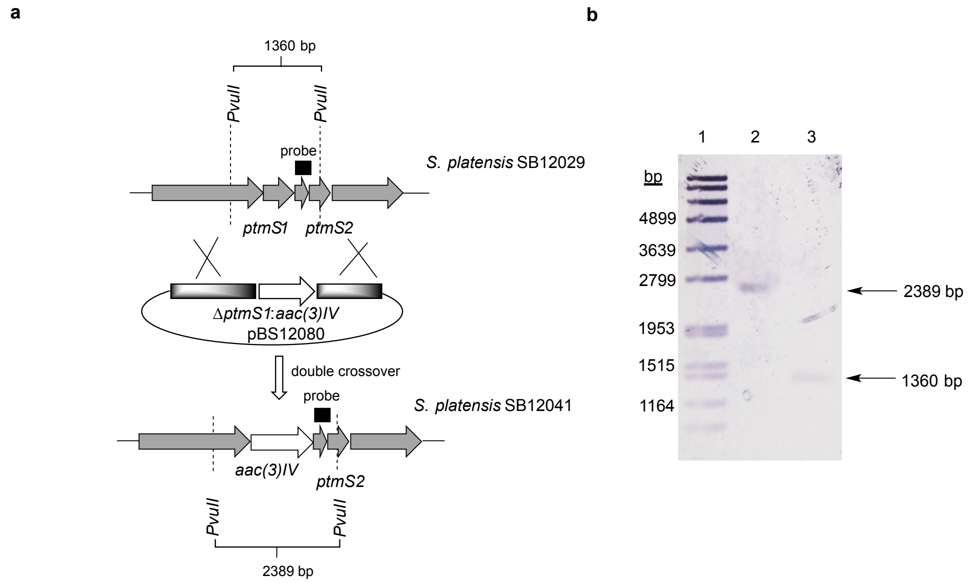

Supplementary Figure 11. Southern analysis of the Δ*ptmR1*/Δ*ptmS1* double mutant *S. platensis* SB12041. **a**, Schematic representation for the deletion of *ptmS1* in *S. platensis* SB12029 by insertion of an *aac(3)IV* + *oriT* cassette. The probe for *ptmS1* (490 bp) was amplified using the primers 739ptmS1south_F and 739ptmS1south_R and genomic DNAs as the template; **b**, Southern blot verification of wild-type *ptmS1* (1360 bp) and double crossover Δ*ptmS1* (2389 bp) mutant genotypes. Lane 1, DNA marker VII, DIG-labeled (Roche); lane 2, *S. platensis* SB12041; lane 3, *S. platensis* SB12029.

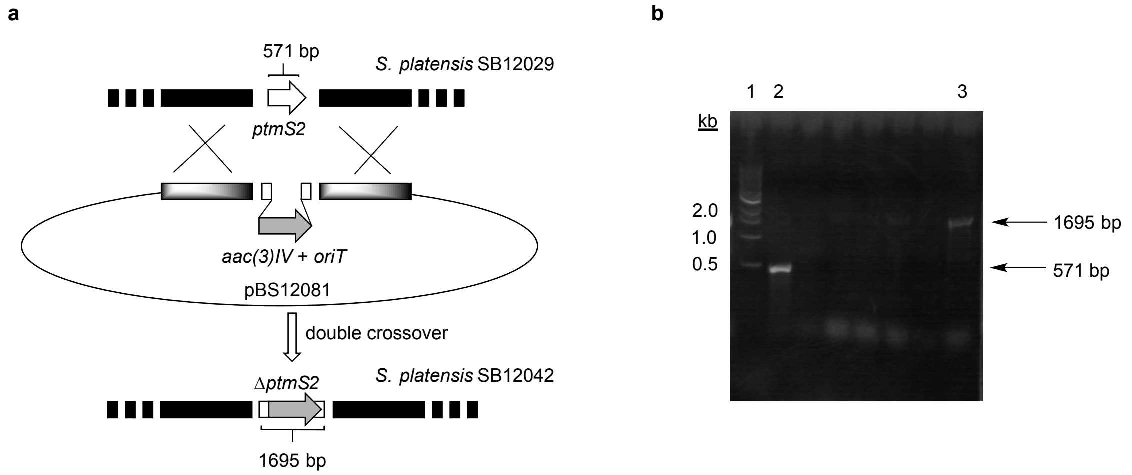

Supplementary Figure 12. Inactivation of *ptmS2* in the PTM-PTN overproducer *S. platensis* SB12029 affording *S. platensis* SB12042. **a**, Schematic representation for the deletion of *ptmS2* in *S. platensis* SB12029 by insertion of an apramycin resistance cassette [*aac(3)IV* + *oriT*]; **b**, PCR verification of wild-type (571 bp) and double crossover (1695 bp) mutant genotypes using the primers 739ptmS2ID_F and 739ptmS2ID_R. Lane 1, 1 kb DNA ladder (NEB); lane 2, *S. platensis* SB12029; lane 3, *S. platensis* SB12042.

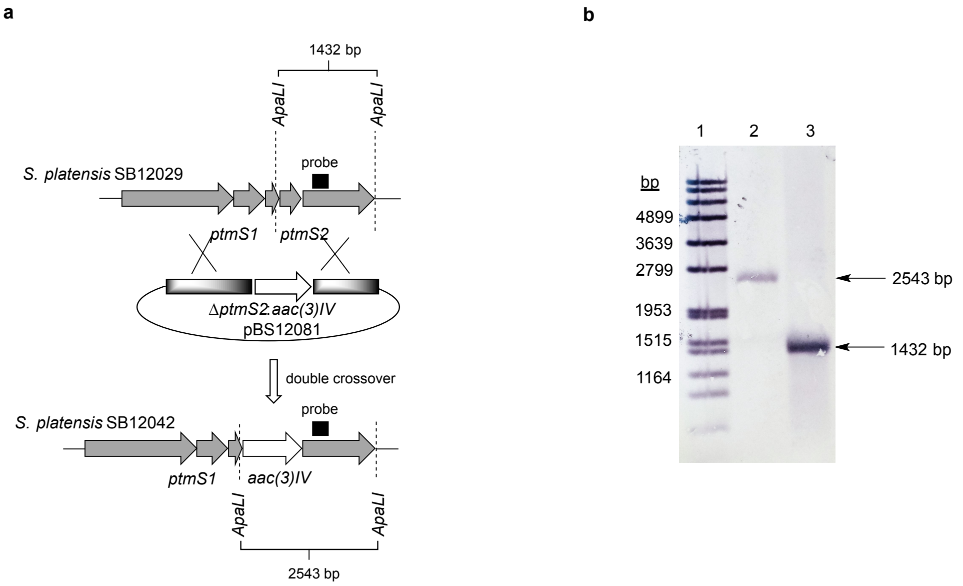

Supplementary Figure 13. Southern analysis of the Δ*ptmR1*/Δ*ptmS2* double mutant *S. platensis* SB12042. **a**, Schematic representation for the deletion of *ptmS2* in *S. platensis* SB12029 by insertion of an *aac(3)IV* + *oriT* cassette. The probe for *ptmS2* (462 bp) was amplified using the primers 739ptmS2south_F and 739ptmS2south_R and genomic DNAs as the template; **b**, Southern blot verification of wild-type *ptmS2* (1432 bp) and double crossover Δ*ptmS2* (2543 bp) mutant genotypes. Lane 1, DNA marker VII, DIG-labeled (Roche); lane 2, *S. platensis* SB12042; lane 3, *S. platensis* SB12029.

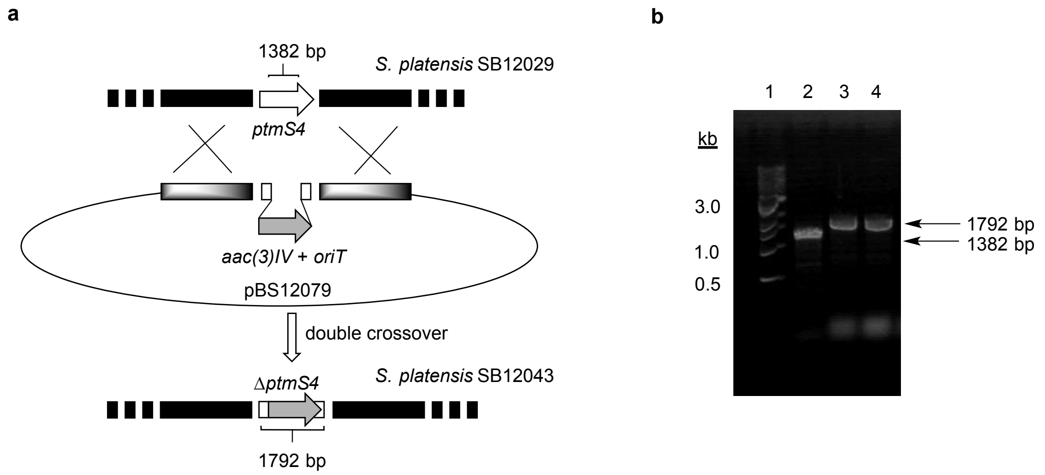

Supplementary Figure 14. Inactivation of *ptmS4* in the PTM-PTN overproducer *S. platensis* SB12029 affording *S. platensis* SB12043. **a**, Schematic representation for the deletion of *ptmS4* in *S. platensis* SB12029 by insertion of an apramycin resistance cassette [*aac(3)IV* + *oriT*]; **b**, PCR verification of wild-type (571 bp) and double crossover (1695 bp) mutant genotypes using the primers 739ptmS4ID_F and 739ptmS4ID_R. Lane 1, 1 kb DNA ladder (NEB); lane 2, *S. platensis* SB12029; lane 3, *S. platensis* SB12043.

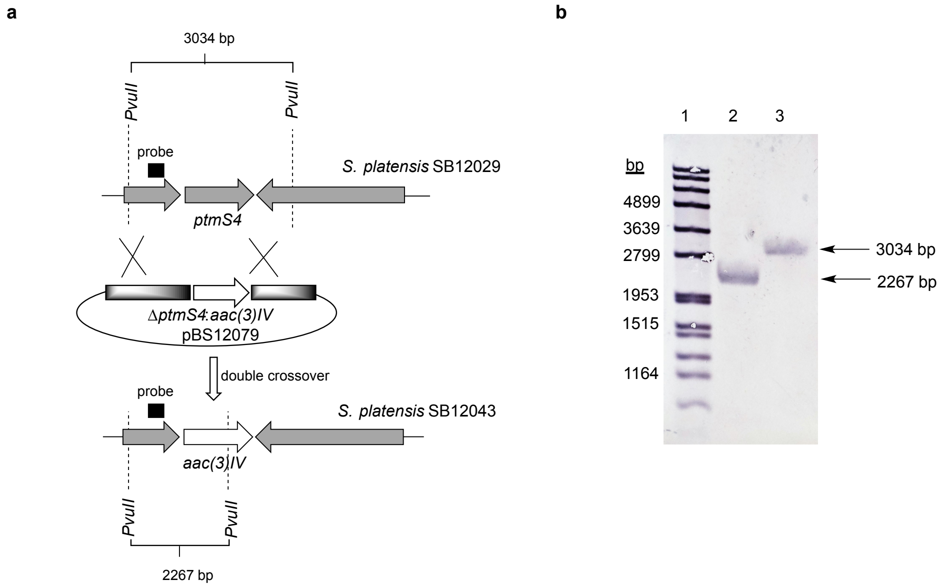

Supplementary Figure 15. Southern analysis of the Δ*ptmR1*/Δ*ptmS4* double mutant *S. platensis* SB12043. **a**, Schematic representation for the deletion of *ptmS4* in *S. platensis* SB12029 by insertion of an *aac(3)IV* + *oriT* cassette. The probe for *ptmS4* (450 bp) was amplified using the primers 739ptmS4south_F and 739ptmS4south_R and genomic DNAs as the template; **b**, Southern blot verification of wild-type *ptmS4* (3043 bp) and double crossover Δ*ptmS4* (2267 bp) mutant genotypes. Lane 1, DNA marker VII, DIG-labeled (Roche); lane 2, *S. platensis* SB12043; lane 3, *S. platensis* SB12029.

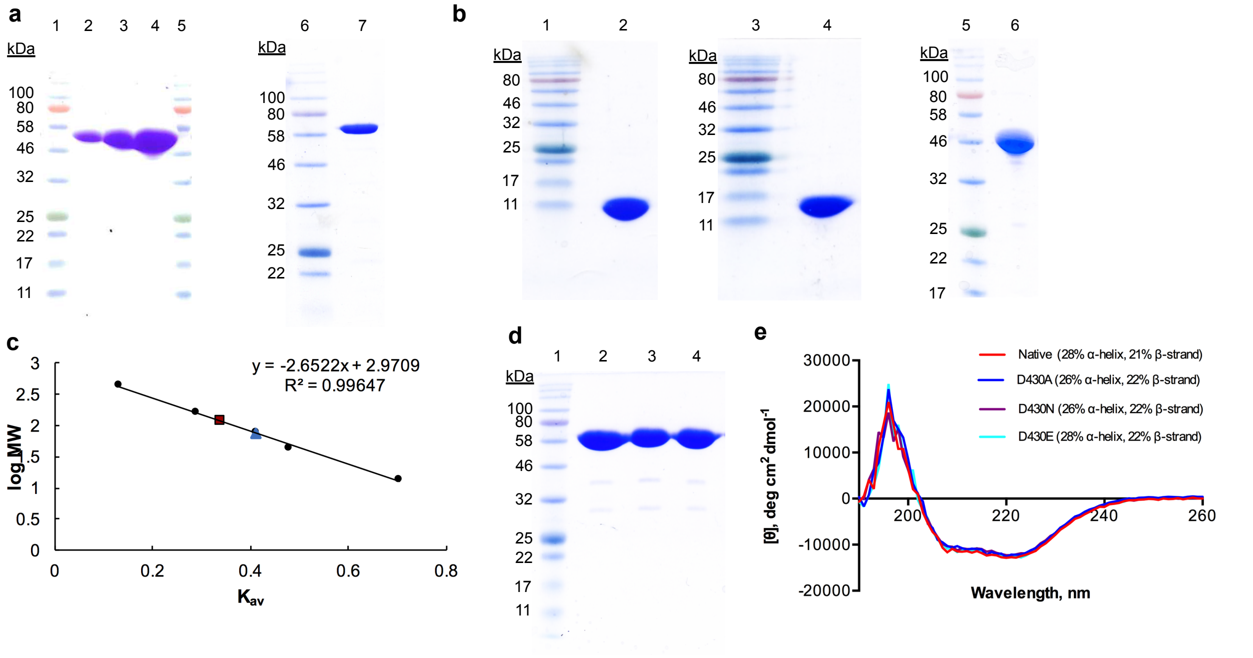

Supplementary Figure 16. SDS-PAGE, size-exclusion chromatography, and far-UV circular dichroism analysis of the purified proteins. **a**, SDS-PAGE gel of purified PtmA3 and PtmU4. Lanes 1, 5, and 6, Color Prestained Protein Standard (NEB); lanes 2 to 4, purified N-His$_6$-PtmA3 (550 amino acids, ~59.5 kDa); lane 7, purified N-His$_6$-PtmU4 (619 amino acids, ~65.2 kDa). **b**, SDS-PAGE gel of purified truncated sulfur-carrier proteins (PtmS2^{GG} and PtmS3^{GG}) and PtmS4. Lanes 1, 3 and 5, Color Prestained Protein Standard (NEB); lane 2, purified truncated N-His$_6$-PtmS2^{GG} (112 amino acids, ~12.4 kDa); lane 4, purified truncated N-His$_6$-PtmS3^{GG} (113 amino acids, ~12.2 kDa); lane 6, purified N-His$_6$-PtmS4 (414 amino acids, ~44.9 kDa). **c**, Size-exclusion chromatography of PtmA3 and PtmU4. PtmA3 (red square) and PtmU4 (blue triangle) eluted at retention volumes of 69.7 and 75.2 mL, correlating to molecular weights (MWs) of 119.0 and 75.9 kDa, respectively. The calculated MWs for PtmA3 and PtmU4 are ~59.5 and ~65.2 kDa, respectively. Thus, PtmA3 and PtmU4 are supported as a dimer and a monomer in solution, respectively. **d**, SDS-PAGE gel of purified PtmU4 mutants. Lane 1, Color Prestained Protein Standard (NEB); lane 2, purified N-His$_6$-PtmU4 D430N; lane 3, purified N-His$_6$-PtmU4 D430E; lane 4, purified N-His$_6$-PtmU4 D430A. **e**, Far-UV circular dichroism (CD) analysis of native PtmU4 and its mutants. The far-UV CD analysis indicated that the secondary structures of PtmU4 mutants were not significantly perturbed relative to native protein.

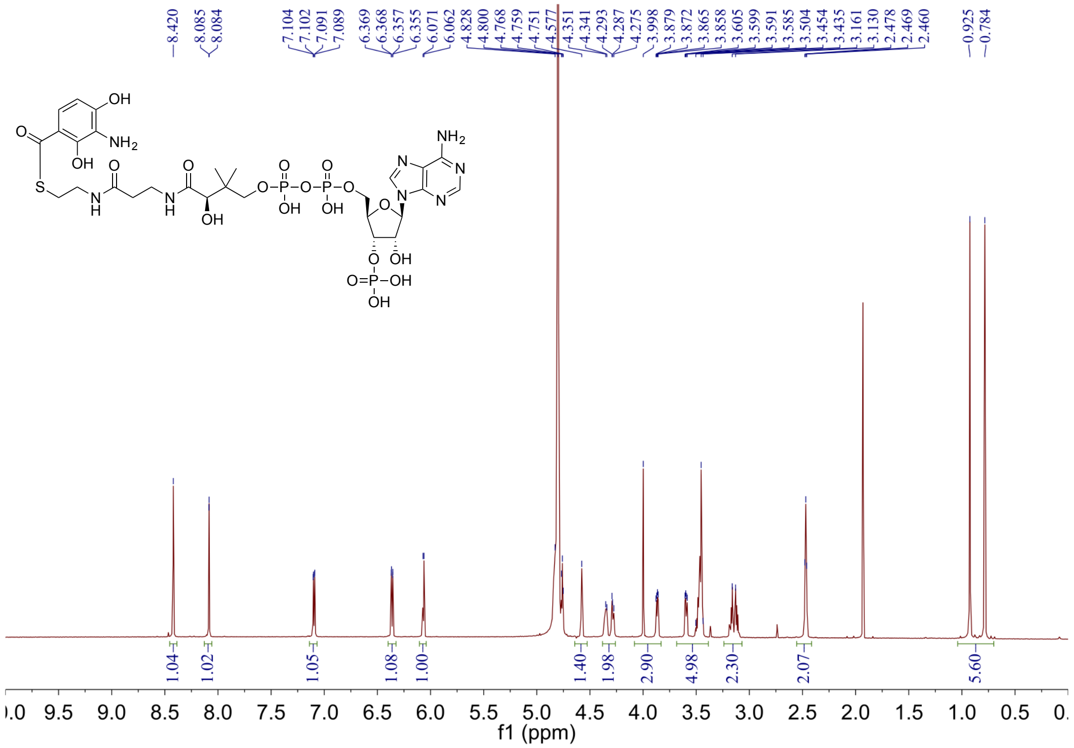

Supplementary Figure 17. ^{1}H NMR spectrum of ADHBCoA (**5-CoA**) in D_2O (700 MHz)

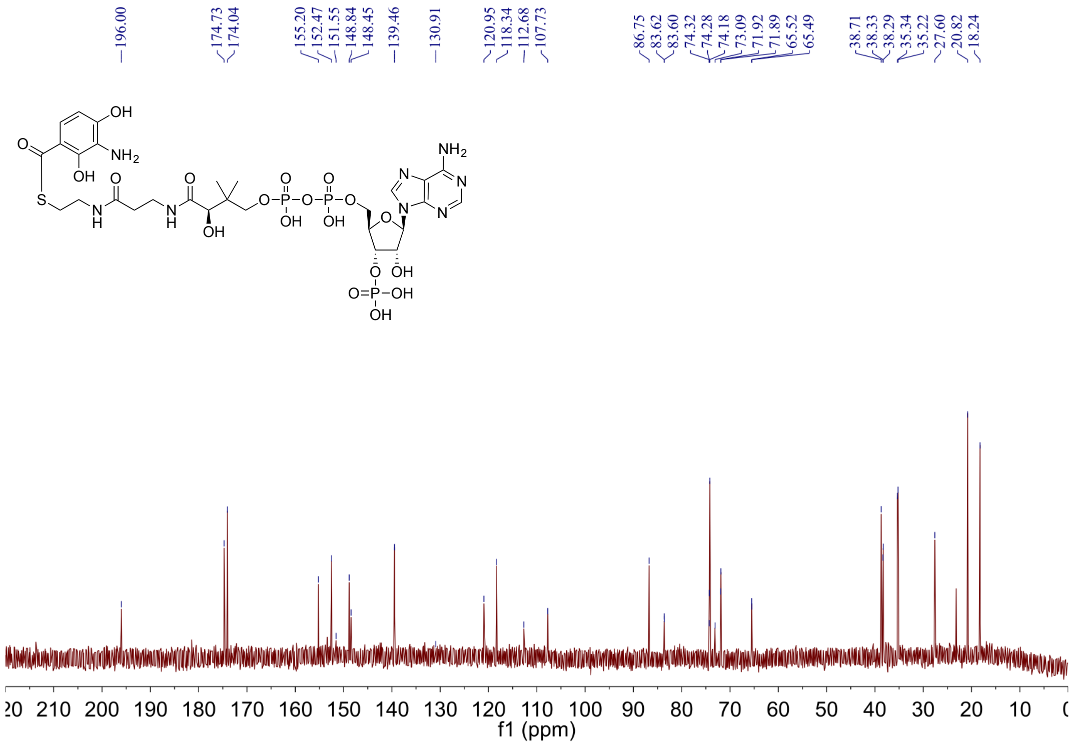

Supplementary Figure 18. ^{13}C NMR spectrum of ADHBCoA (**5-CoA**) in D_2O (175 MHz)

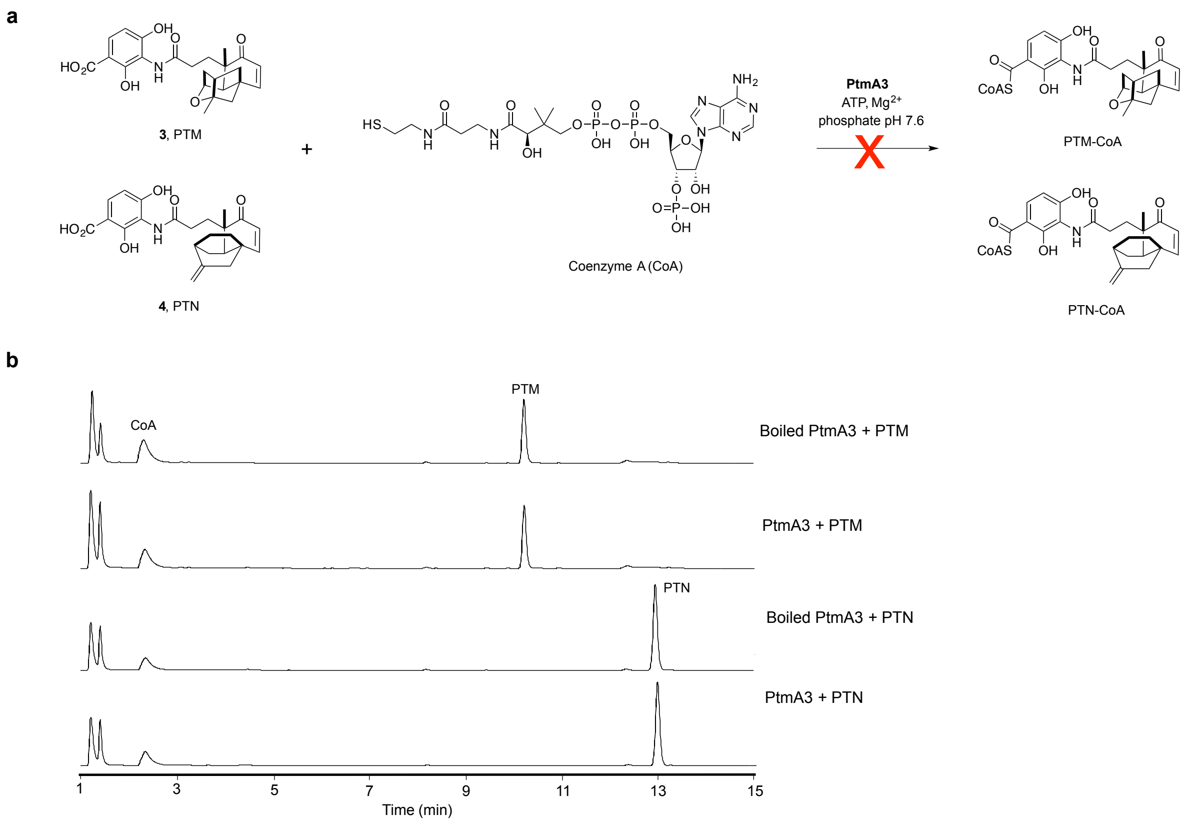

Supplementary Figure 19. HPLC chromatograms of PtmA3 reaction with platensimycin (**3**, PTM) and platencin (**4**, PTN). **a**, Chemical drawing of the PtmA3 reaction with PTM and PTN; **b**, HPLC chromatograms of the PtmA3 reaction with PTM and PTN using the UV detection at λ 280 nm.

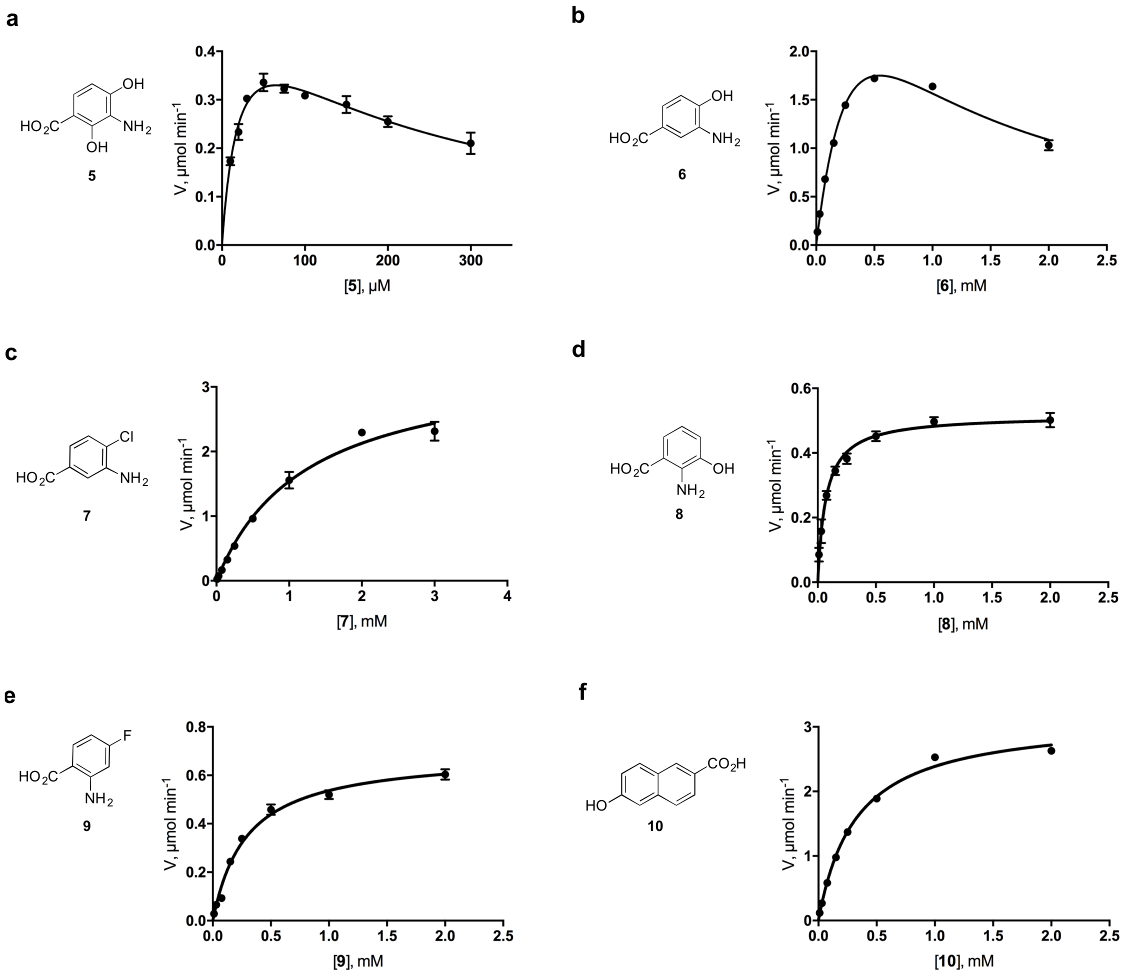

Supplementary Figure 20. Steady-state kinetics of PtmA3. Non-linear Michaelis-Menten regression plots of rate vs substrate concentrations were generated using GraphPad Prism 7 software (La Jolla, CA) to calculate the kinetic constants for each substrate. The determined kinetic parameters are summarized in Fig. 3b in the main text. Kinetics were performed in triplicate and each data point represents the mean of the three independent assays with error bars representing the standard deviation. **a**, PtmA3 (40 nM) was incubated with varying concentrations (10–300 µM) of **5**. **b**, PtmA3 (200 nM) was incubated with varying concentrations (0.01–2 mM) of **6**. **c**, PtmA3 (500 nM) was incubated with varying concentrations (0.01–3 mM) of **7**. **d**, PtmA3 (500 nM) was incubated with varying concentrations (0.01–2 mM) of **8**. **e**, PtmA3 (500 nM) was incubated with varying concentrations (0.01–2 mM) of **9**. **f**, PtmA3 (200 nM) was incubated with varying concentrations (0.01–2 mM) of **10.**

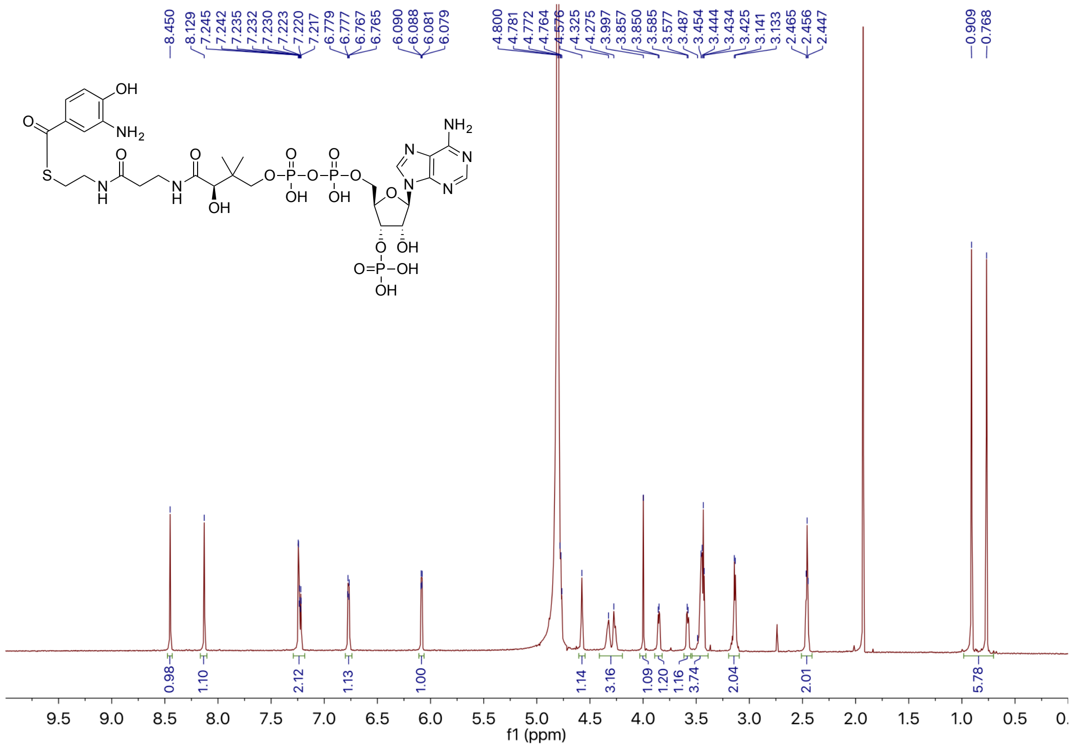

Supplementary Figure 21. ^{1}H NMR spectrum of **6-CoA** in D_2O (700 MHz)

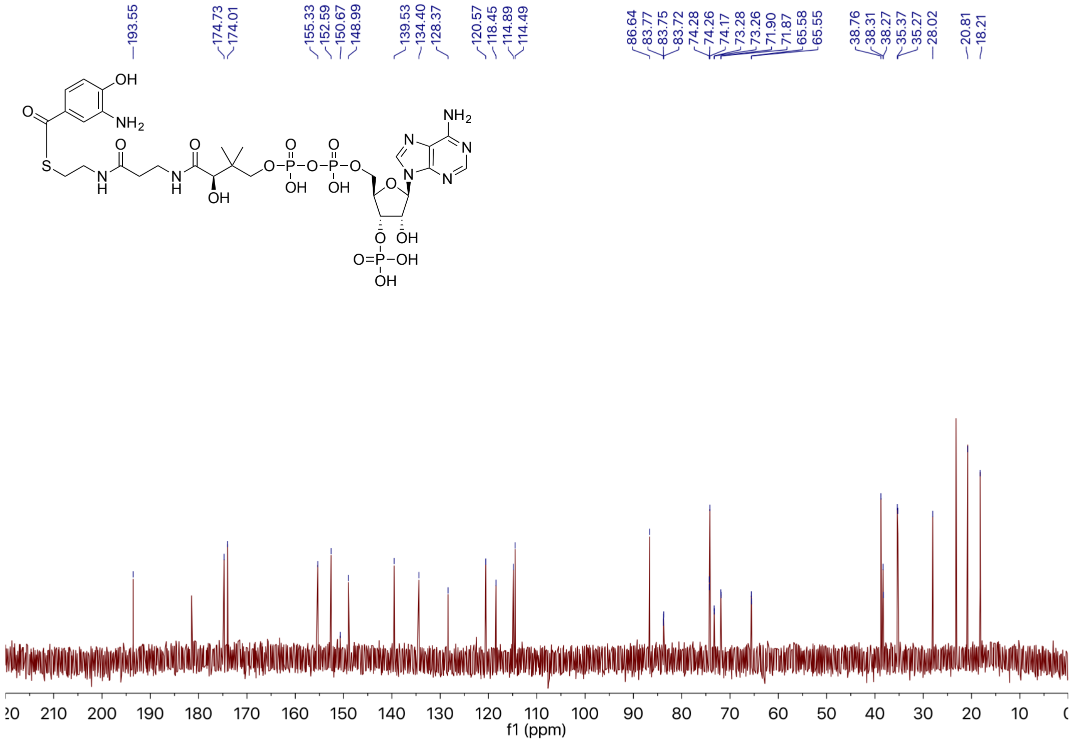

Supplementary Figure 22. ^{13}C NMR spectrum of **6-CoA** in D_2O (175 MHz)

a

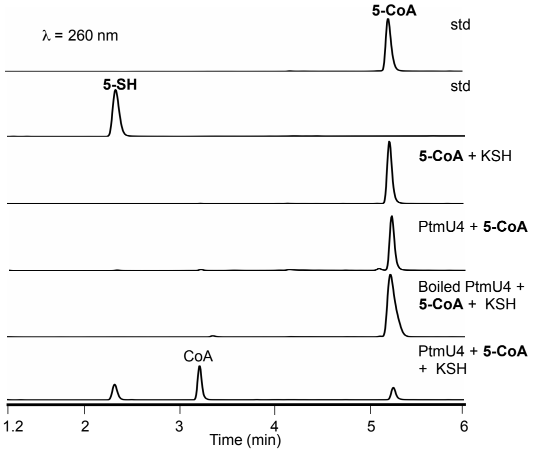

b

PtmU4 Variant	Rate (s^{-1})	Relative Rate (%)
Native	(2.4 ± 0.07) x 10^{-3}	100 ± 3
D430A	(1.1 ± 0.02) x 10^{-5}	0.46 ± 0.07
D430E	(5.5 ± 0.2) x 10^{-6}	0.23 ± 0.08
D430N	(2.0 ± 0.01) x 10^{-5}	0.81 ± 0.05

Supplementary Figure 23. *in vitro* reactions of PtmU4 and its mutants. **a**, UV at 260 nm from HPLC analysis of *in vitro* PtmU4 reactions with **5-CoA** using potassium hydrosulfide (KSH) as a sulfur donor surrogate. std, standard. **b**, Relative activities of PtmU4 mutants. All experiments were performed in triplicate and the data are listed with standard deviations. The relative rates are compared to the native protein.

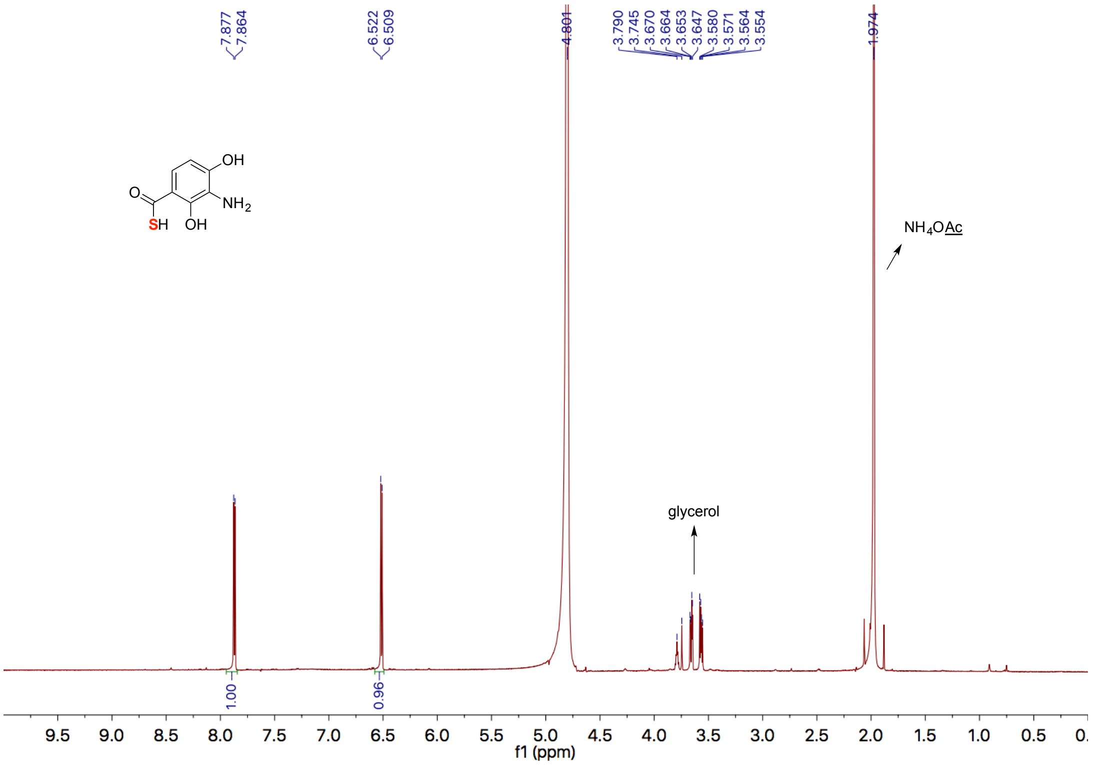

Supplementary Figure 24. ^{1}H NMR spectrum of ADHBSH (**5-SH**) in D_2O (700 MHz)

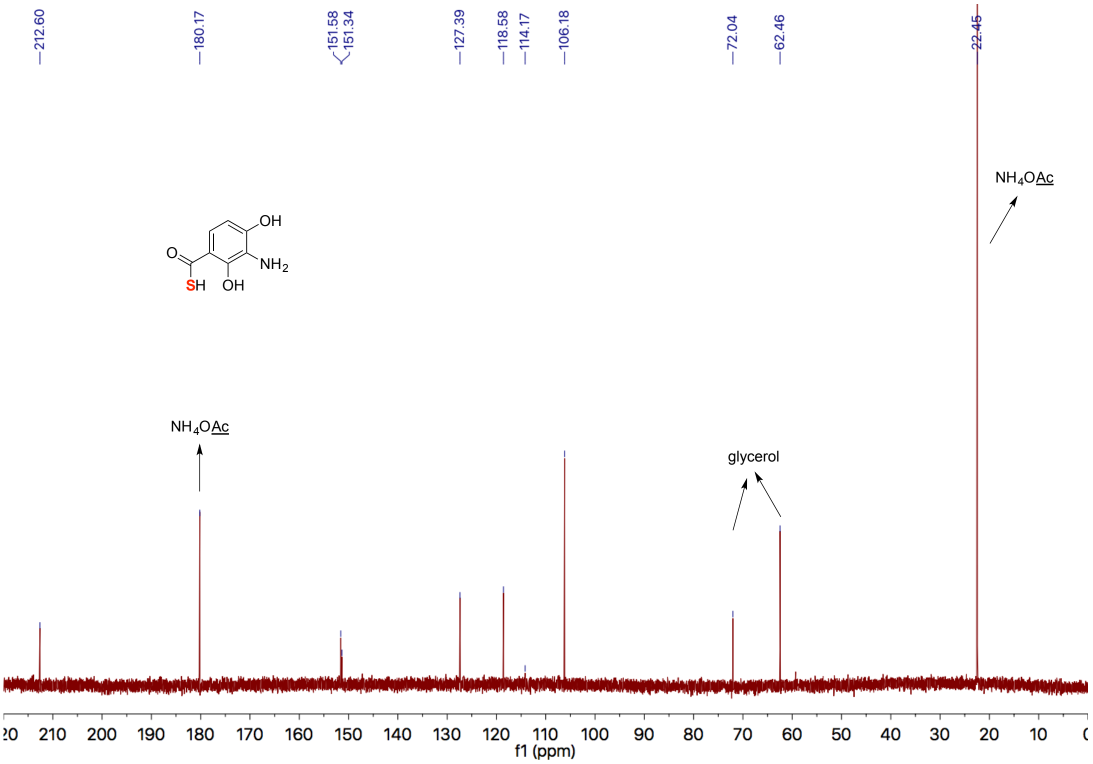

Supplementary Figure 25. ^{13}C NMR spectrum of ADHBSH (**5-SH**) in D_2O (175 MHz)

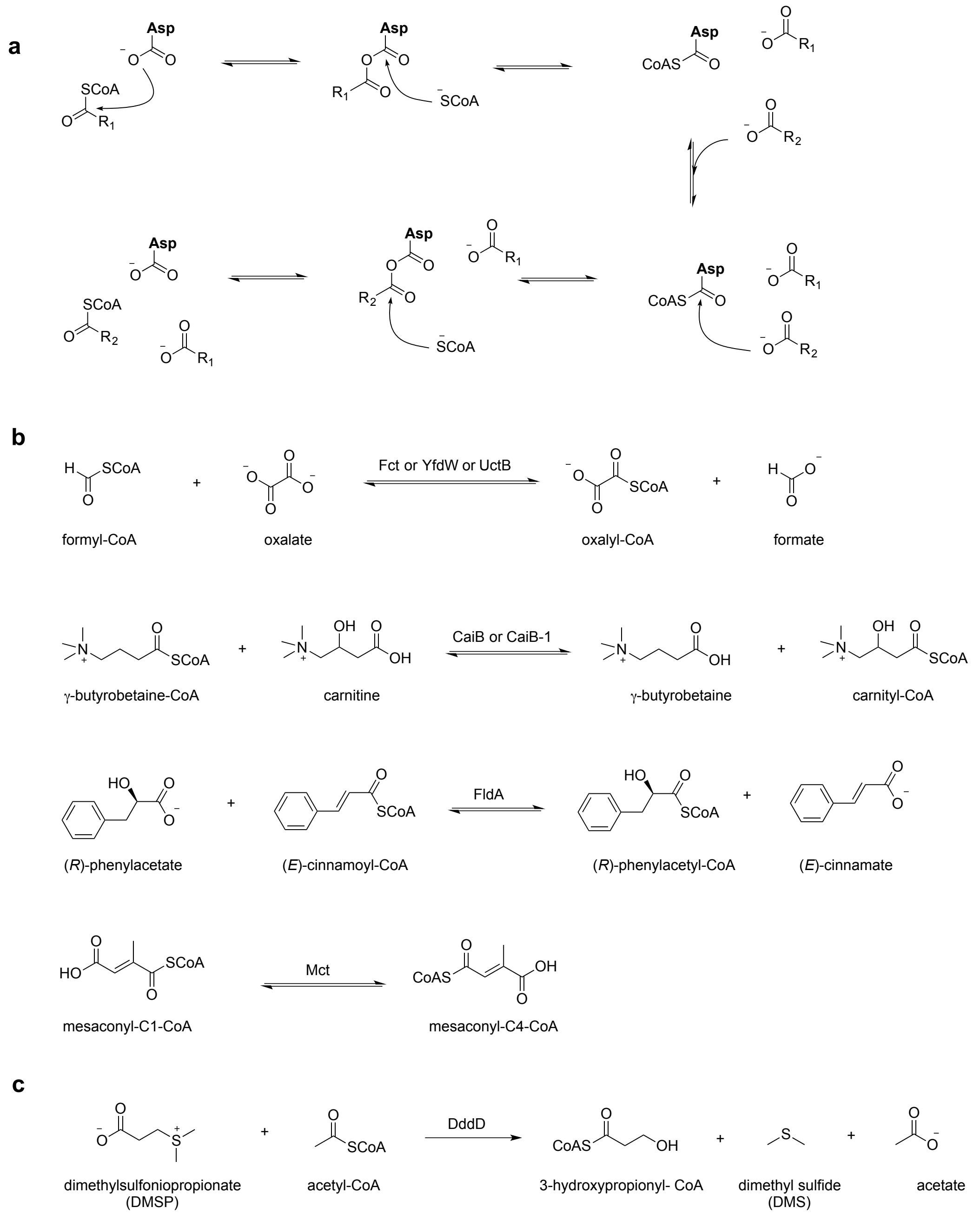

Supplementary Figure 26. General mechanism and selected examples of type III CoA-transferases. **a**, General mechanism of CoA transfer of one-domain type III CoA-transferases. **b**, Reversible CoA transfer reactions performed by one-domain type III CoA-transferases; Mct catalyzes an intramolecular transfer of CoA. **c**, The reaction of dimethyl sulfide release performed by the only known two-domain type III CoA-transferase, DddD[21,22].

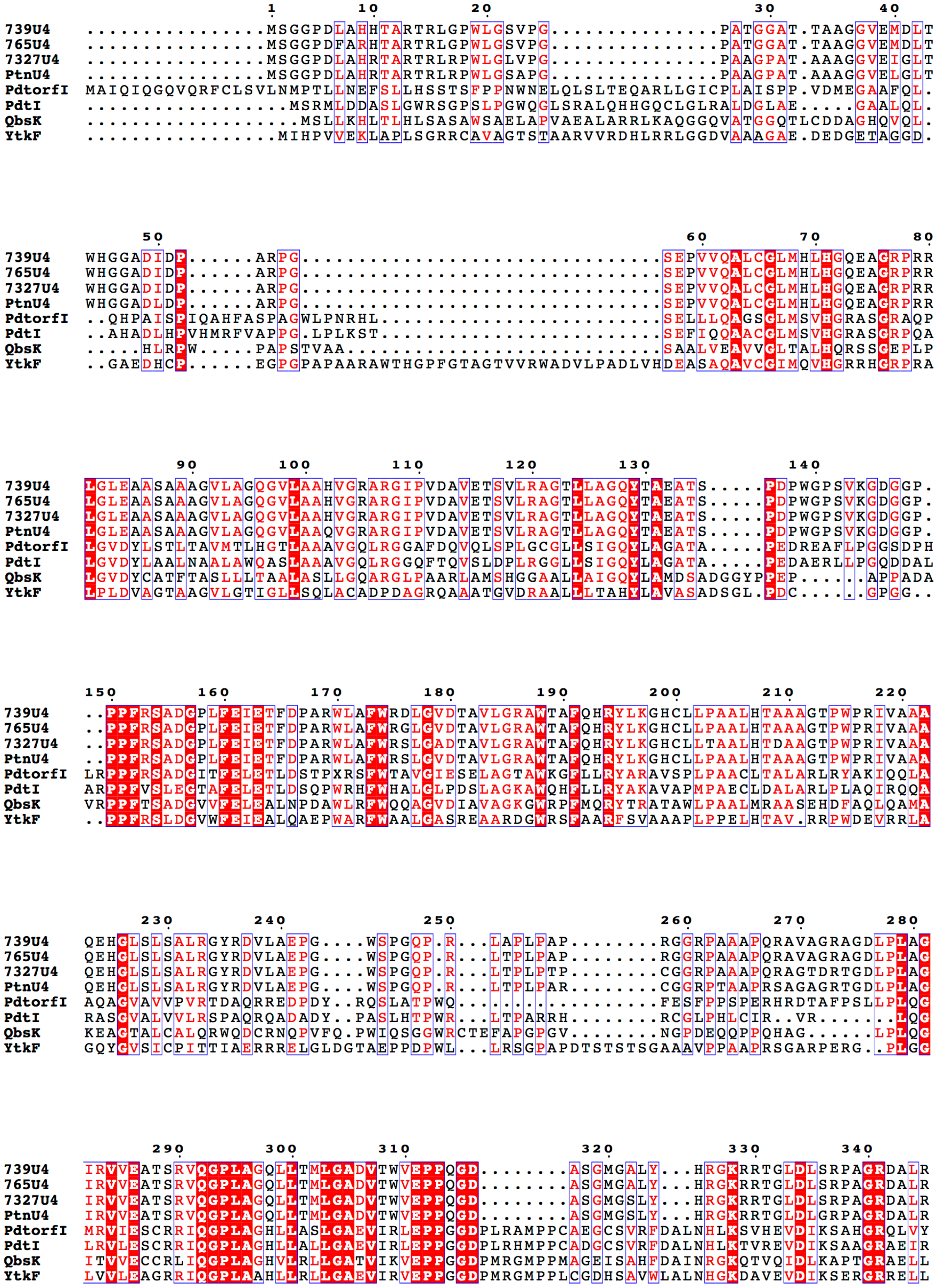

Supplementary Figure 27. To be continued

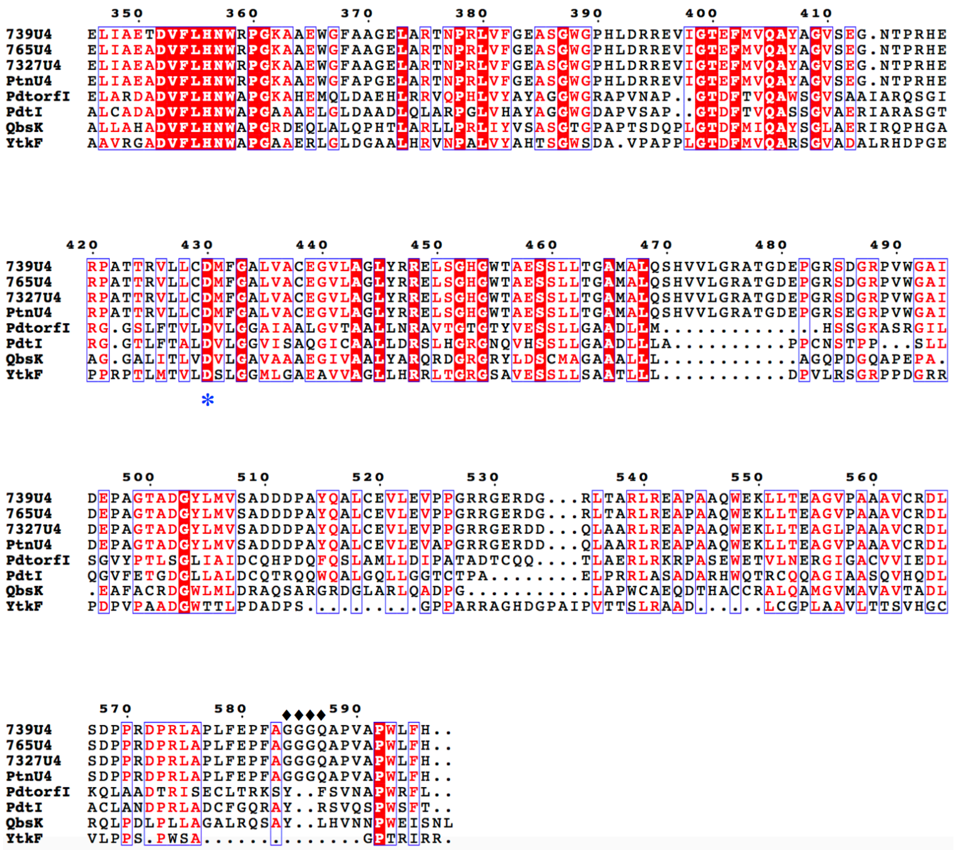

Supplementary Figure 27. Sequence alignment of selected PtmU4 homologues from bacteria. Aligned residues are colored based on the level of conservation (red box with white character shows strict identity, red character similarity, and blue frame similarity across groups). The conserved catalytic residue, aspartic acid (D430), is shown with blue asterisks. The glycine loop (GGGQ, indicated with black diamonds), which was reported playing a central role during catalysis in formyl-CoA transferase (Frc)[23], was not conserved in all two-domain CoA-transferases and only found in PtmU4/PtnU4 homologues. The alignment was created with MUSCLE[19] and rendered with ESPript 3.0[20].

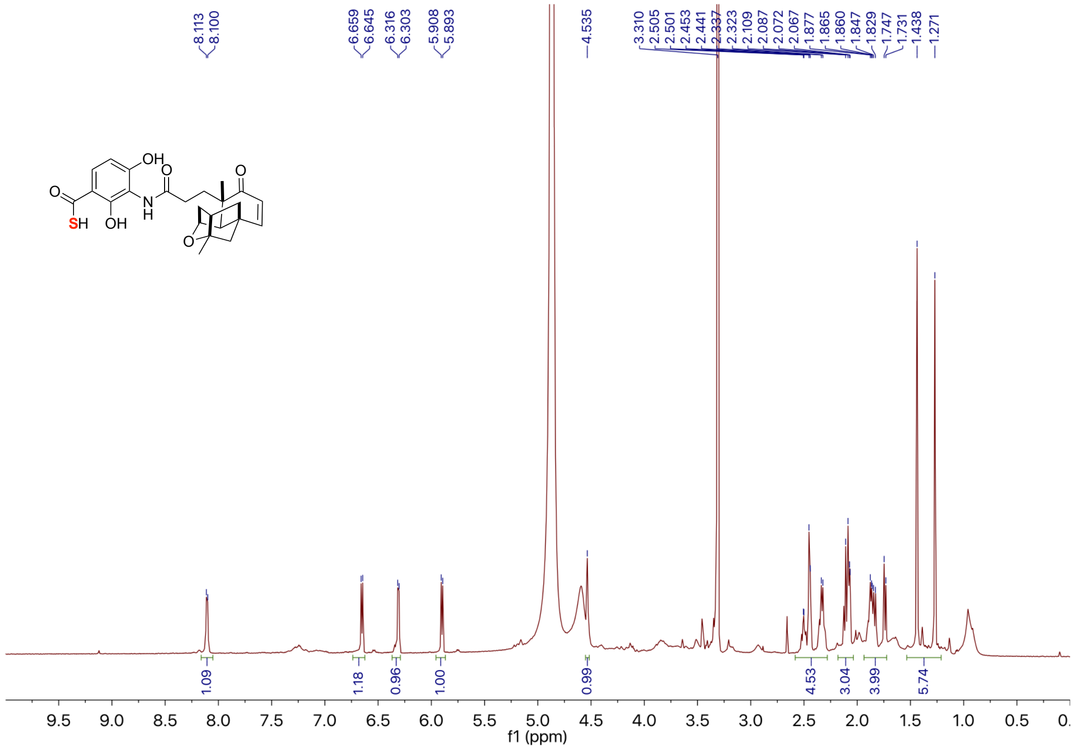

Supplementary Figure 28. ^{1}H NMR spectrum of thioPTM (**1**) in CD_3OD (700 MHz)

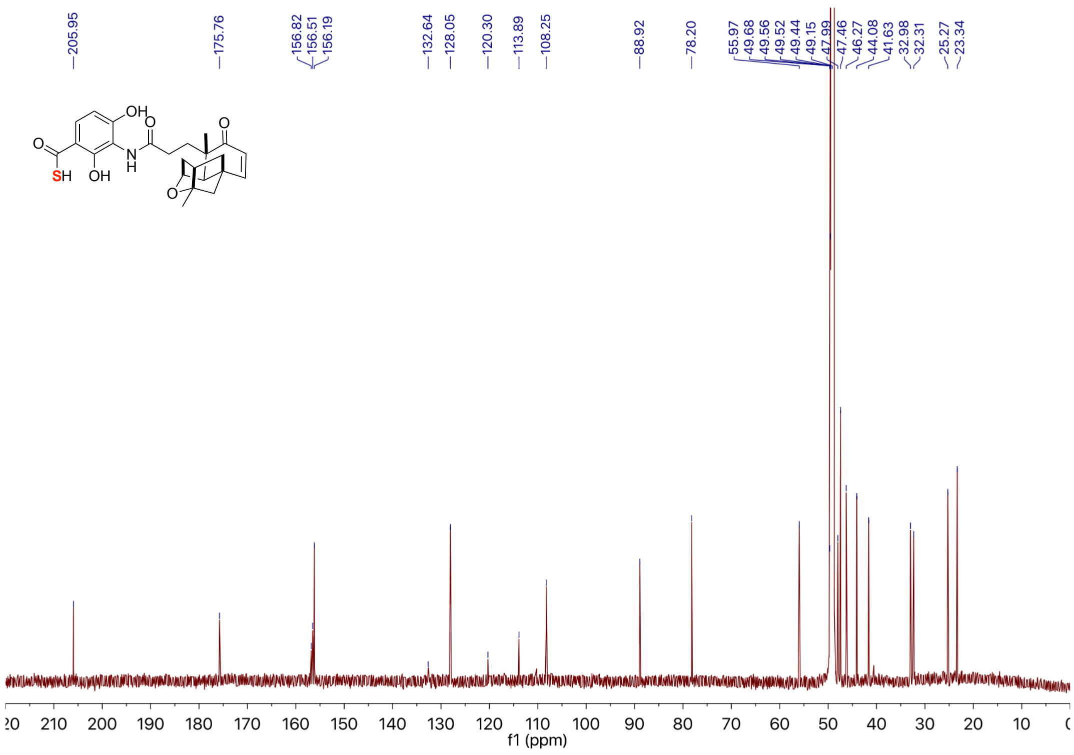

Supplementary Figure 29. ^{13}C NMR spectrum of thioPTM (**1**) in CD_3OD (175 MHz)

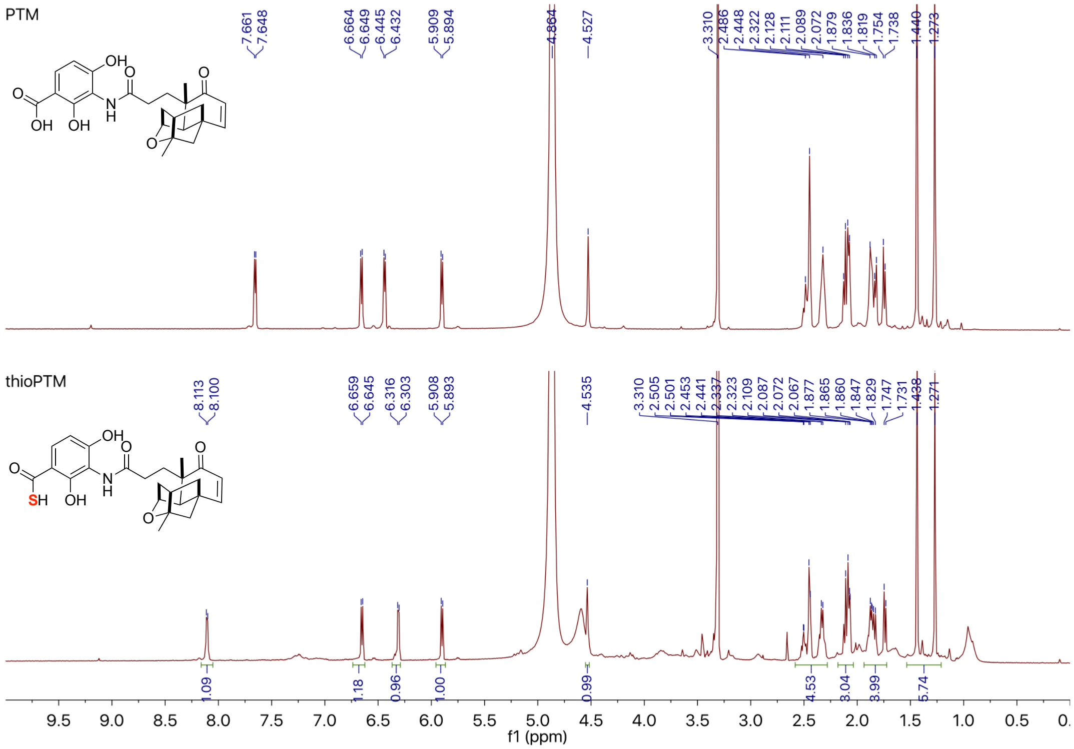

Supplementary Figure 30. Comparison of the ^{1}H NMR spectra of PTM (**3**) and thioPTM (**1**)

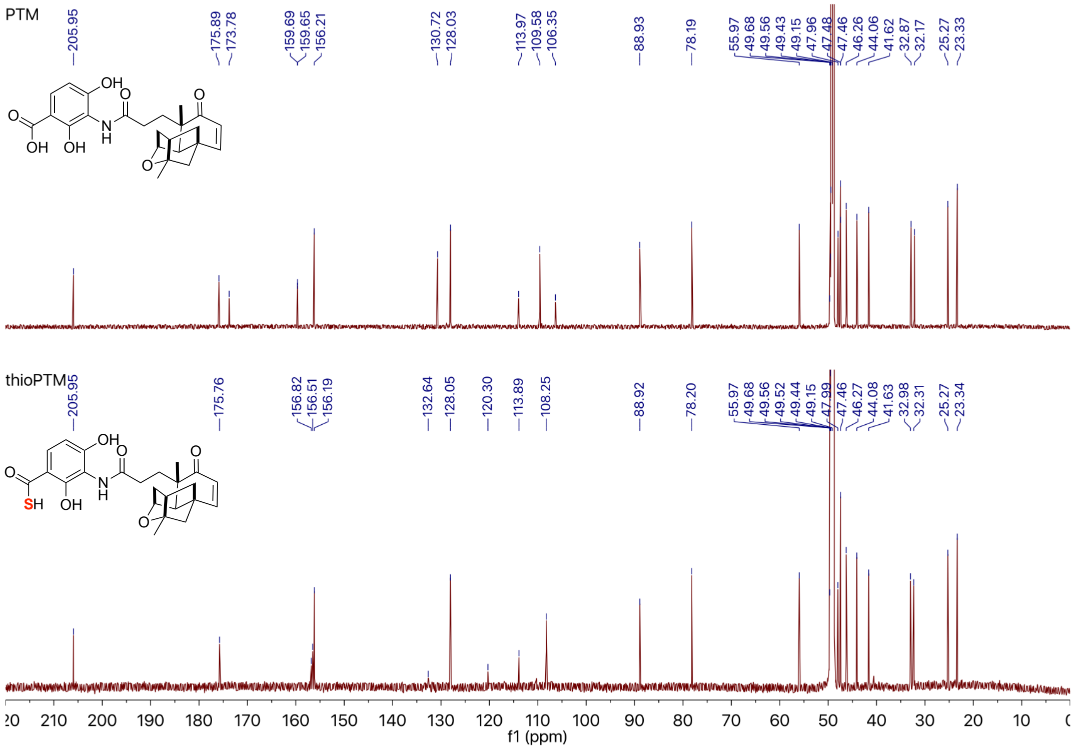

Supplementary Figure 31. Comparison of the ^{13}C NMR spectra of PTM (**3**) and thioPTM (**1**)

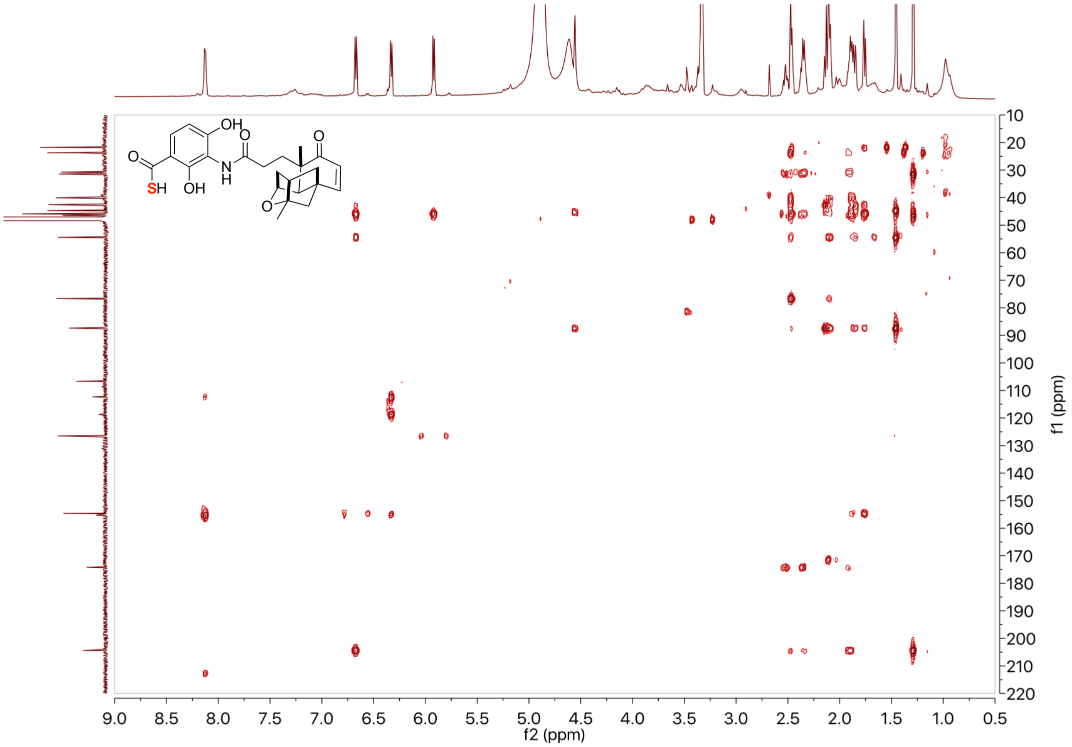

Supplementary Figure 32. ^{1}H–^{13}C HMBC spectrum of thioPTM (**1**) in CD_3OD

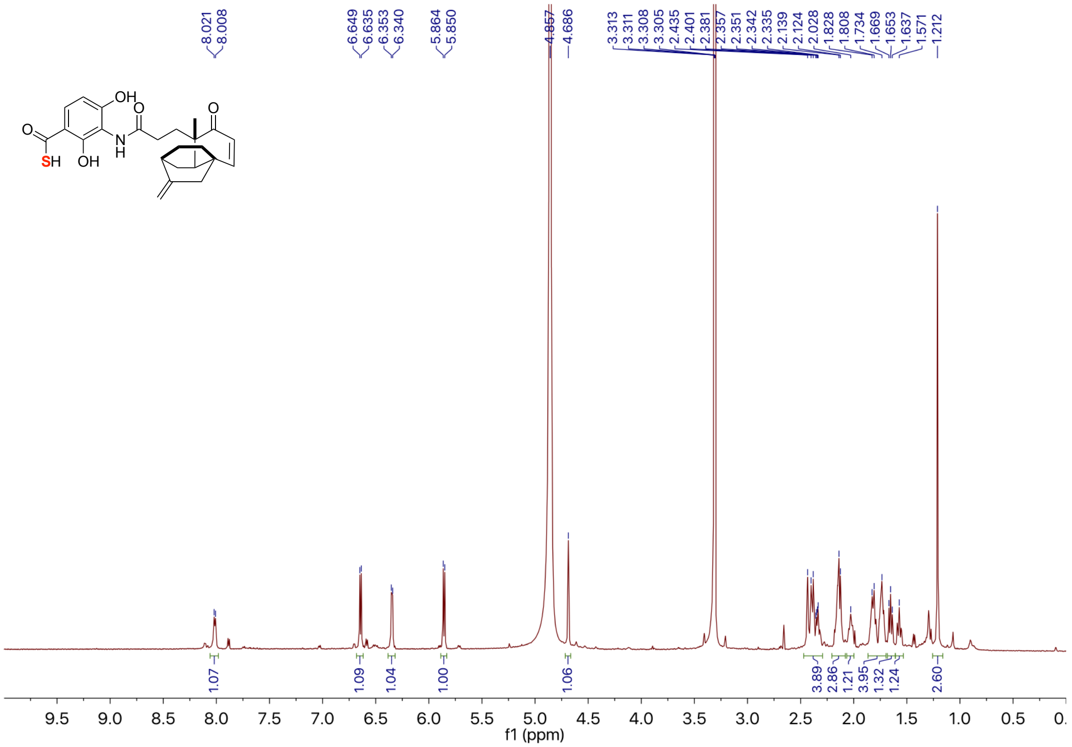

Supplementary Figure 33. ^{1}H NMR spectrum of thioPTN (**2**) in CD_3OD (700 MHz)

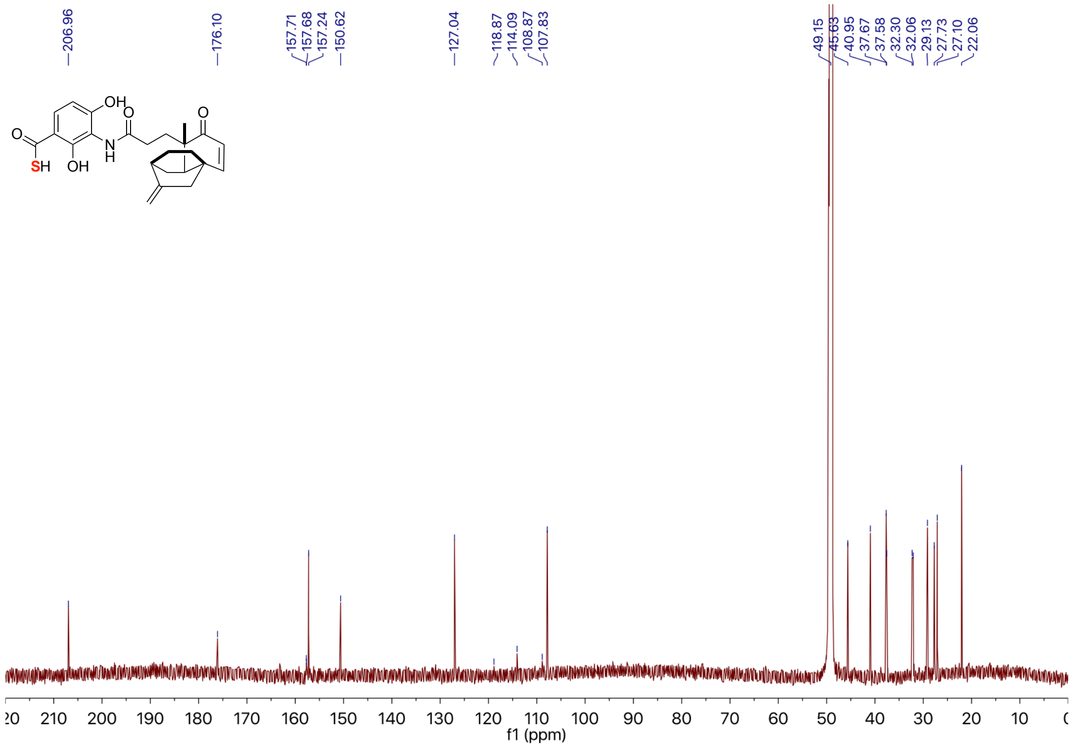

Supplementary Figure 34. ^{13}C NMR spectrum of thioPTN (**2**) in CD_3OD (175 MHz)

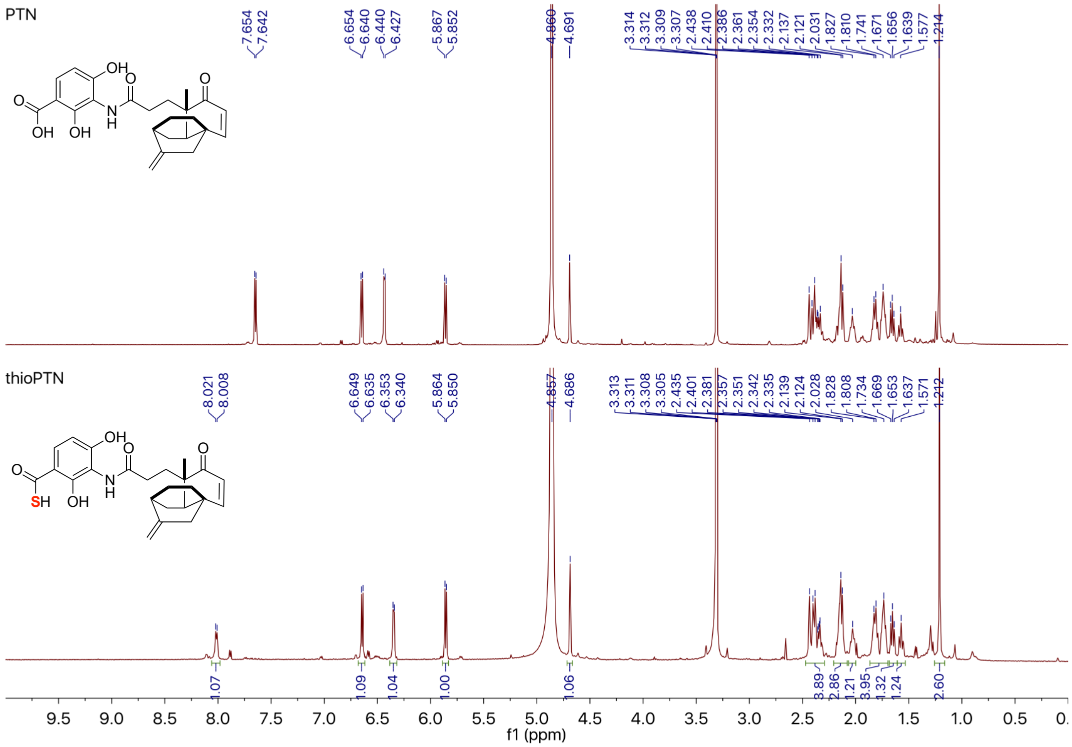

Supplementary Figure 35. Comparison of the ^{1}H NMR spectra of PTN (**4**) and thioPTN (**2**)

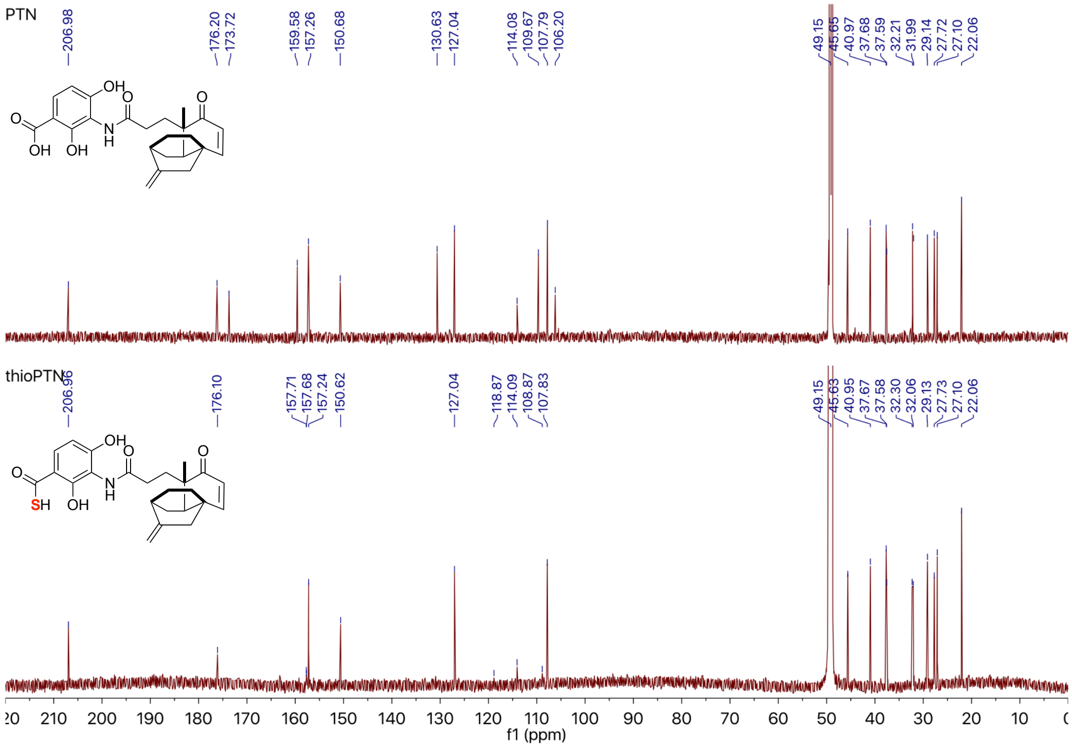

Supplementary Figure 36. Comparison of the ^{13}C NMR spectra of PTN (**4**) and thioPTN (**2**)

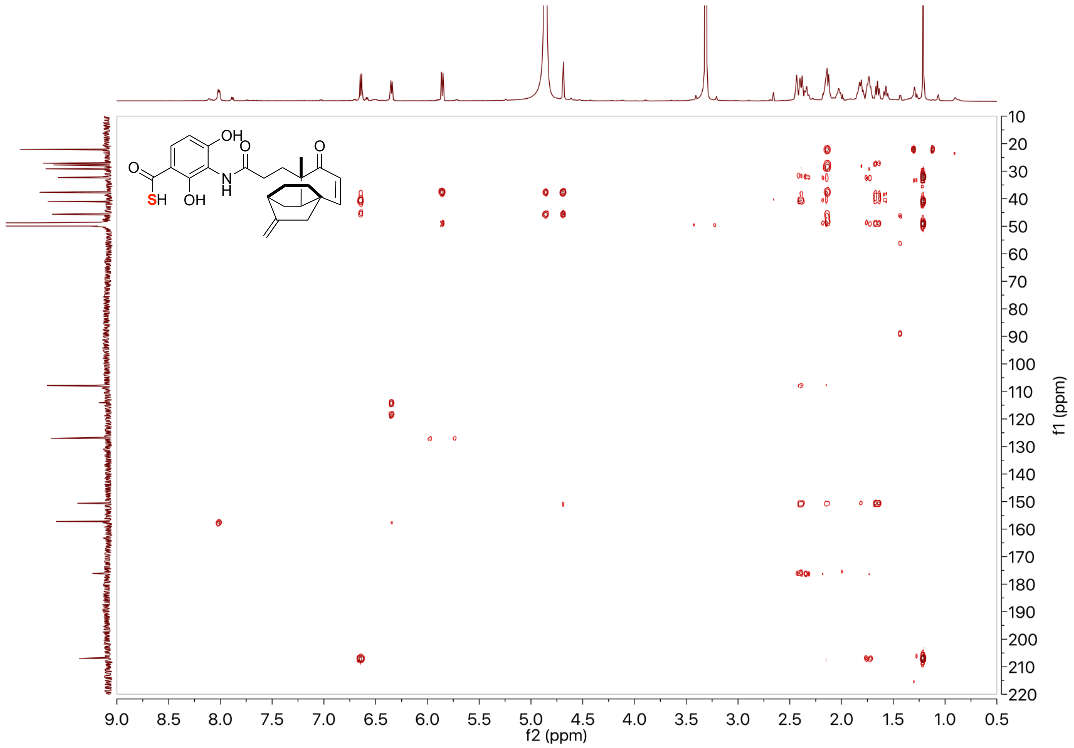

Supplementary Figure 37. ^1H–^{13}C HMBC spectrum of thioPTN (**2**) in CD_3OD

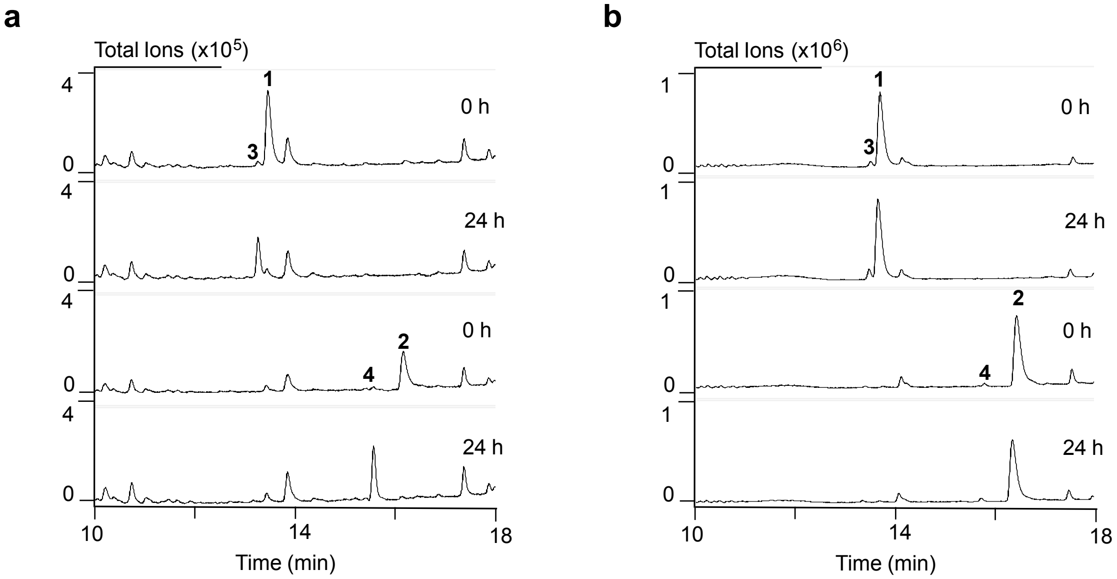

Supplementary Figure 38. In vitro stability of thioPTM and thioPTN. **a**, Total ion chromatogram (TIC) from LC-MS analysis of **1** and **2** in Muller-Hinton (MH) broth (antibacterial assay medium). Both compounds are unstable in MH broth at 37 ^{o}C: more than 80% of **1** and 90% of **2** were hydrolyzed to **3** and **4**, respectively, after a 24 h incubation. Detection of **3** and **4** confirms that **3** and **4** are non-enzymatic hydrolyzed products of **1** and **2**, respectively. **b**, TIC analysis of thioPTM and thioPTN in HBS-P+ buffer (SPR experimental buffer). Both compounds are stable in HBS-P+ buffer at room temperature: less than 5% thioPTM (**1**) and thioPTN (**2**) were hydrolyzed to PTM (**3**) and PTN (**4**), respectively, after a 24 h incubation. Liquid chromatography for LC-MS analysis was performed using an 18 min solvent gradient (0.4 mL min^{-1}) from 5% – 100% CH_3OH in H_2O containing 0.1% formic acid on an Agilent Poroshell 120 EC-C18 column (50 mm × 4.6 mm, 2.7 μm), which is identical to the method used in the Supplementary Figure 2c. Although the elution retention times of **3** and **4** are comparable, the elution retention times of **1** and **2** were ~0.7 min later than those in Supplementary Figure 2c, suggesting that **1** and **2** may adopt their thiocarboxylate forms in MH broth and HBS-P+ buffer.

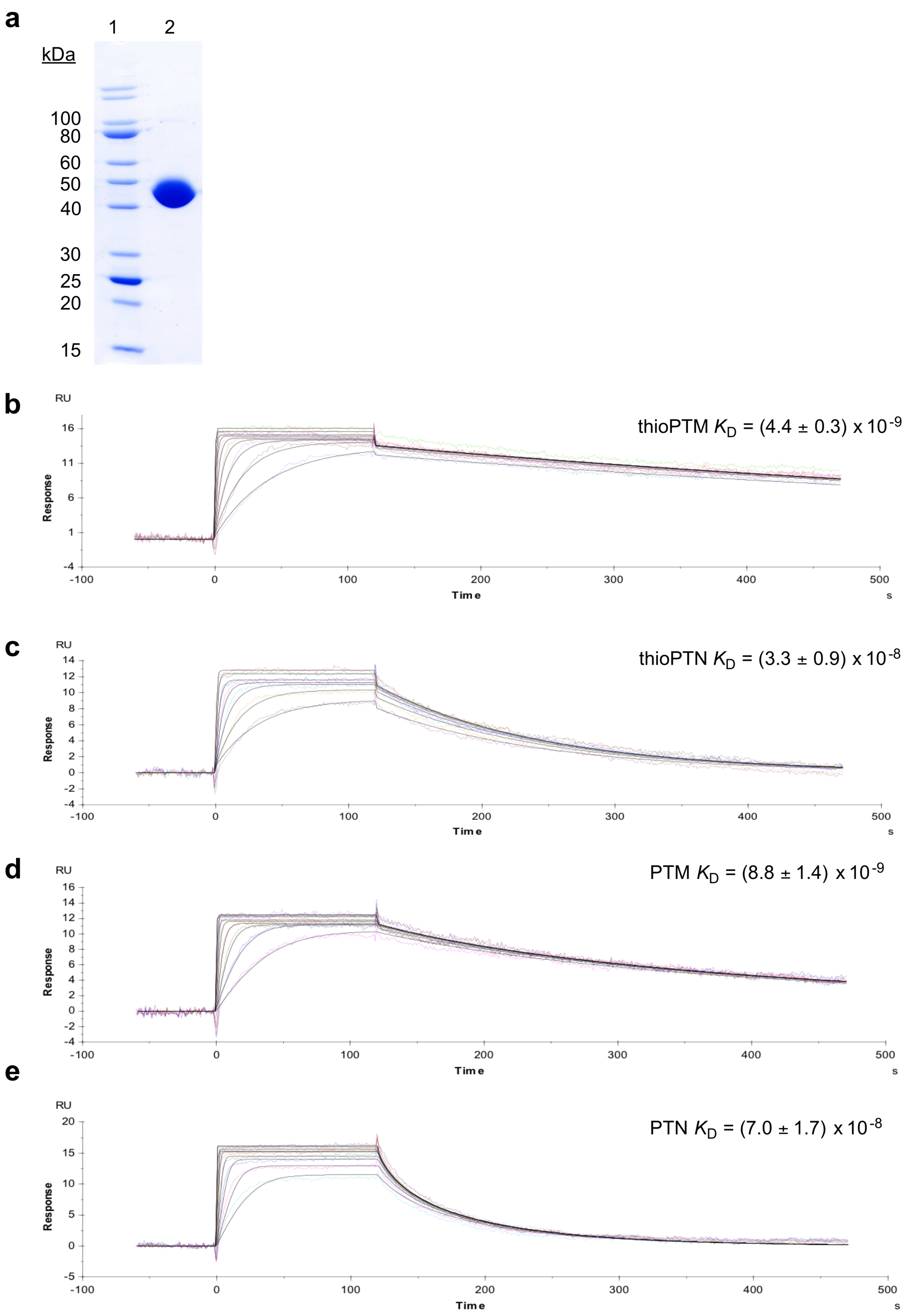

Supplementary Figure 39. Binding kinetics of compounds 1–4 with *E. coli* FabF C163Q. **a**, SDS-PAGE gel of purified *E. coli* FabF C163Q. Lane 1, Unstained Protein Ladder, Broad Range (NEB); lane 2, purified N-His$_6$-FabF C163Q (436 amino acids, ~45.7 kDa). Representative SPR sensorgrams for **b**, thioPTM (**1**); **c**, thioPTN (**2**); **d**, PTM (**3**); and **e**, PTN (**4**). Colored curves depict experimental data at different analyte (**1–4**) concentrations (0, 0.078, 0.156, 0.313, 0.625, 1.25, 2.5, 5, and 10 μM) with globally fitted curves overlaid in black.

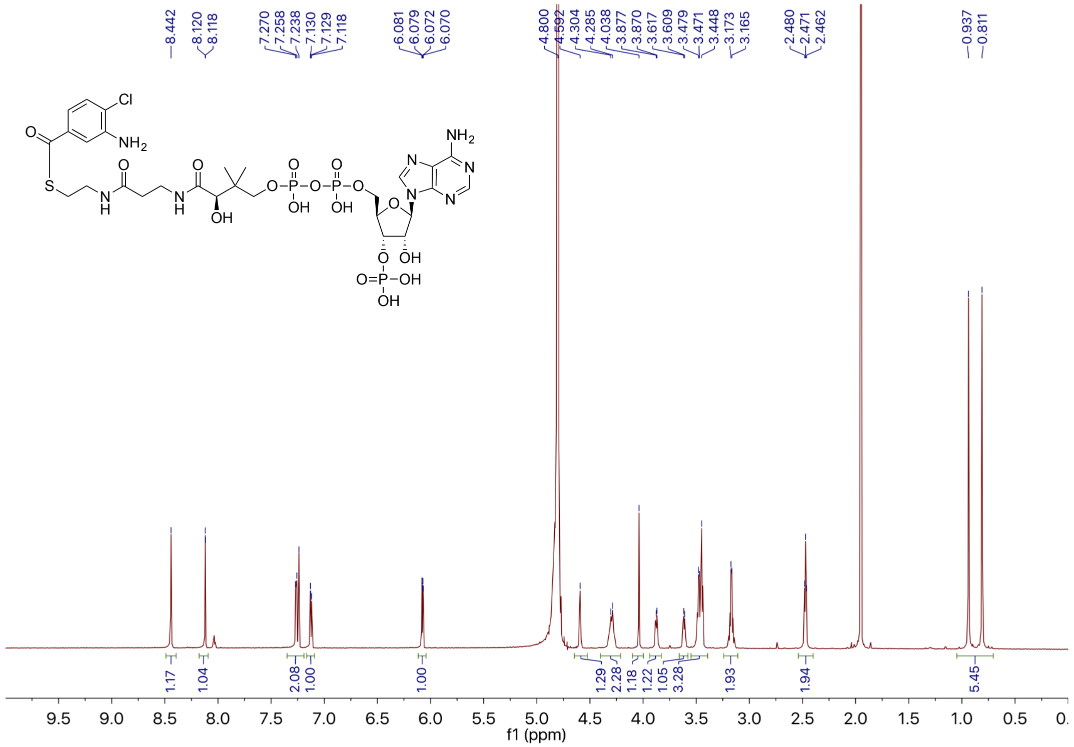

Supplementary Figure 40. ^{1}H NMR spectrum of **7-CoA** in D_2O (700 MHz)

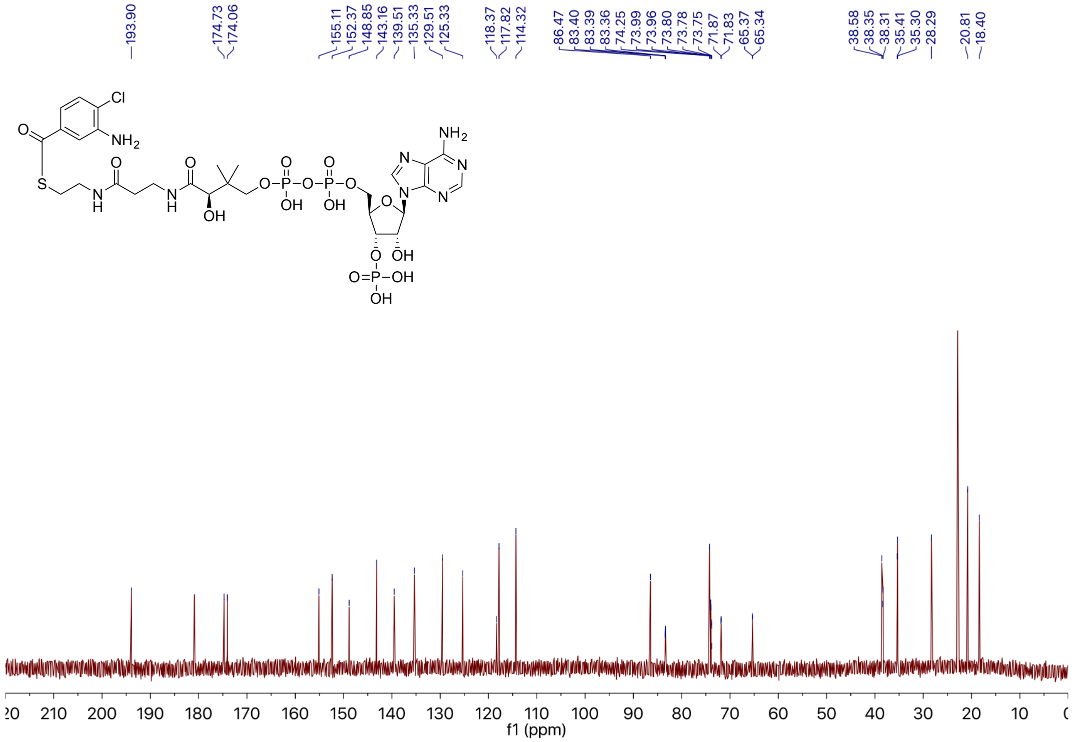

Supplementary Figure 41. ^{13}C NMR spectrum of **7-CoA** in D_2O (175 MHz)

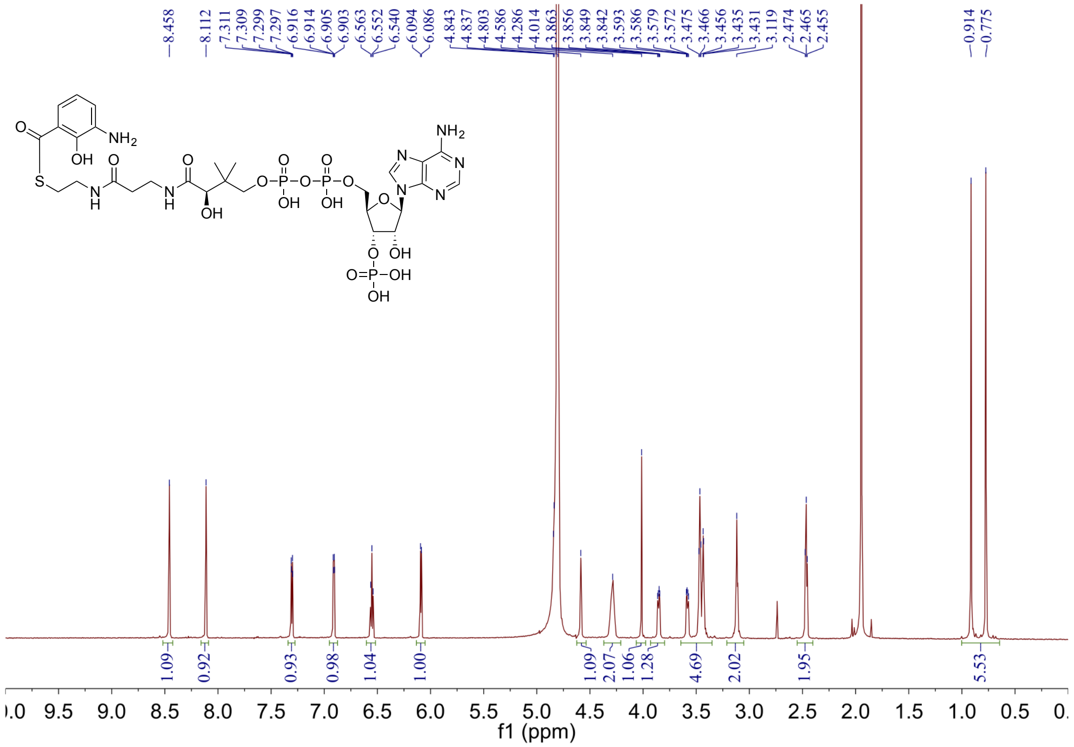

Supplementary Figure 42. ^{1}H NMR spectrum of **8-CoA** in D_2O (700 MHz)

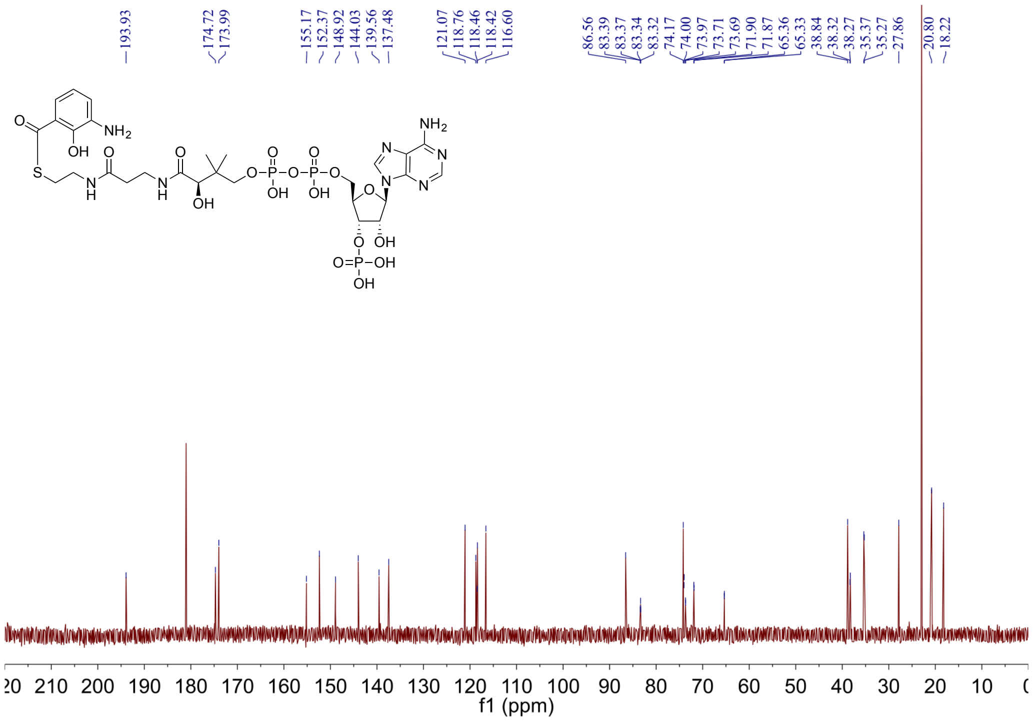

Supplementary Figure 43. ^{13}C NMR spectrum of **8-CoA** in D_2O (175 MHz)

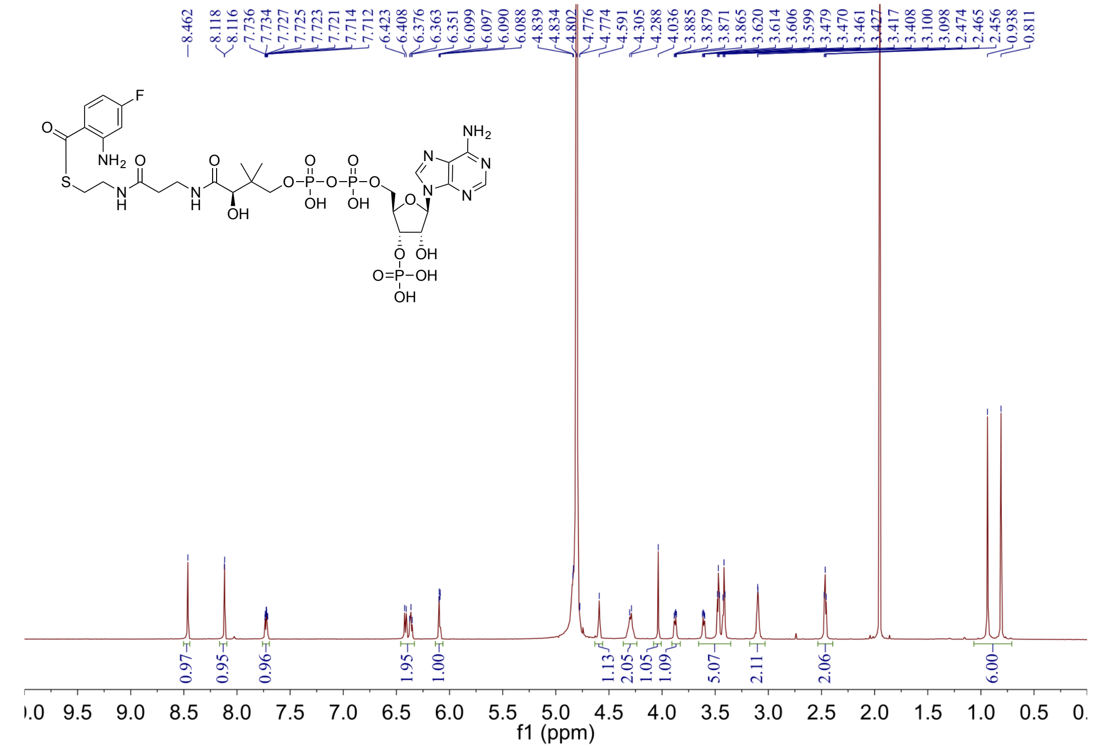

Supplementary Figure 44. ^{1}H NMR spectrum of **9-CoA** in D_2O (700 MHz)

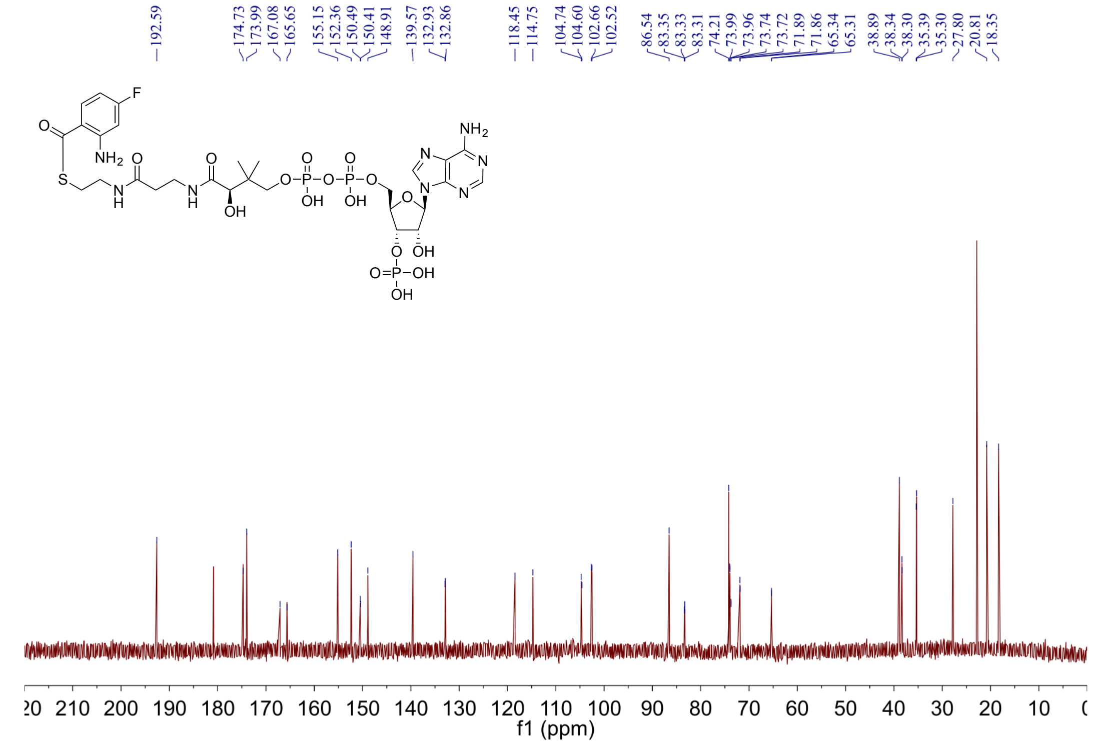

Supplementary Figure 45. ^{13}C NMR spectrum of **9-CoA** in D_2O (175 MHz)

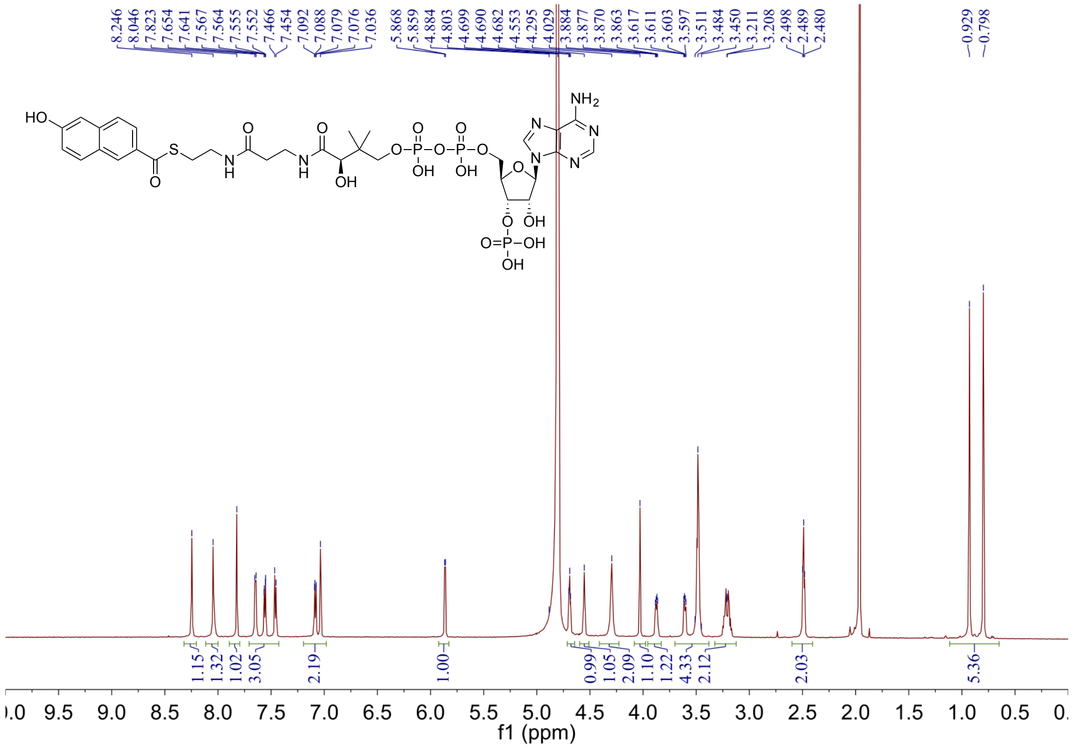

Supplementary Figure 46. ^{1}H NMR spectrum of **10-CoA** in D_2O (700 MHz)

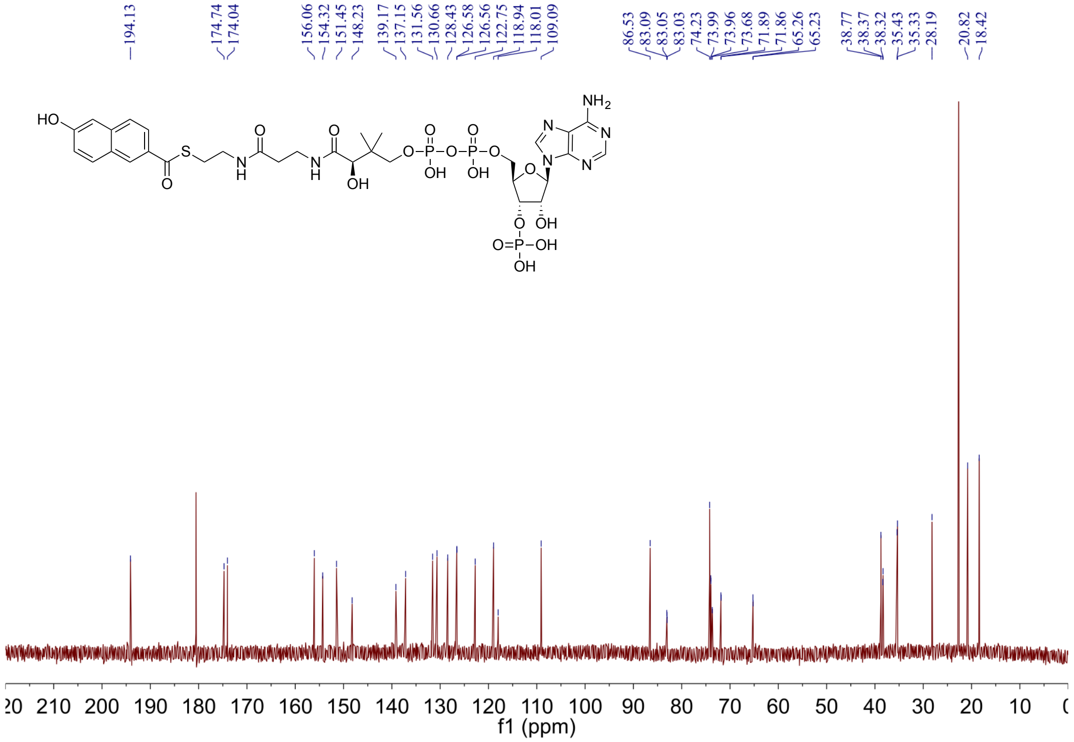

Supplementary Figure 47. ^{13}C NMR spectrum of **10-CoA** in D_2O (175 MHz)

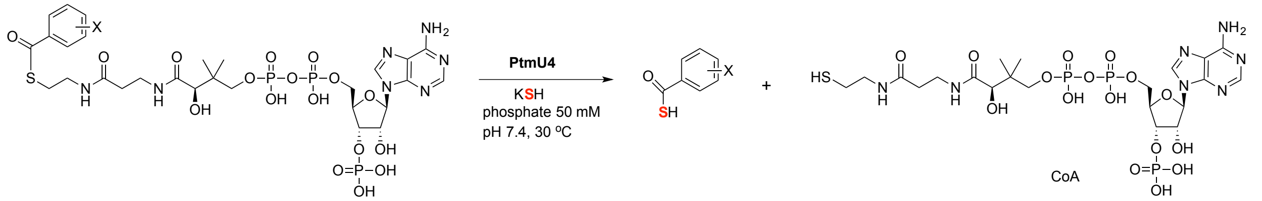

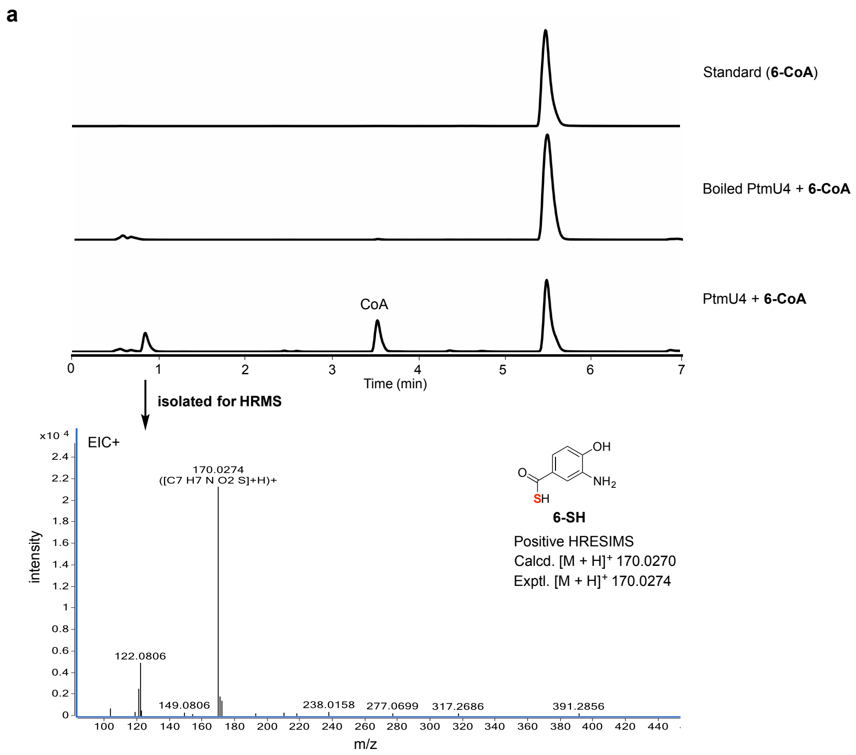

Supplementary Figure 48. To be continued

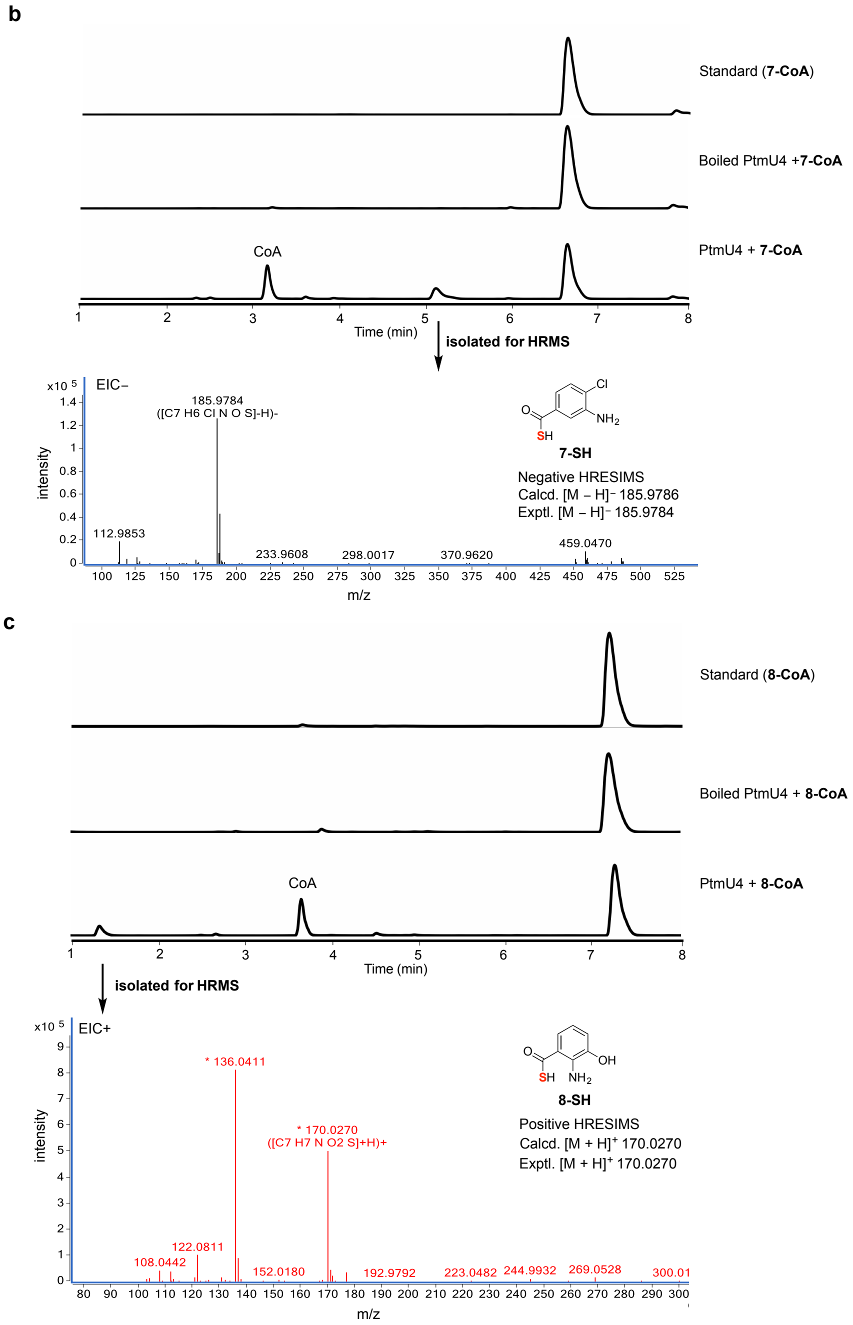

Supplementary Figure 48. To be continued

d

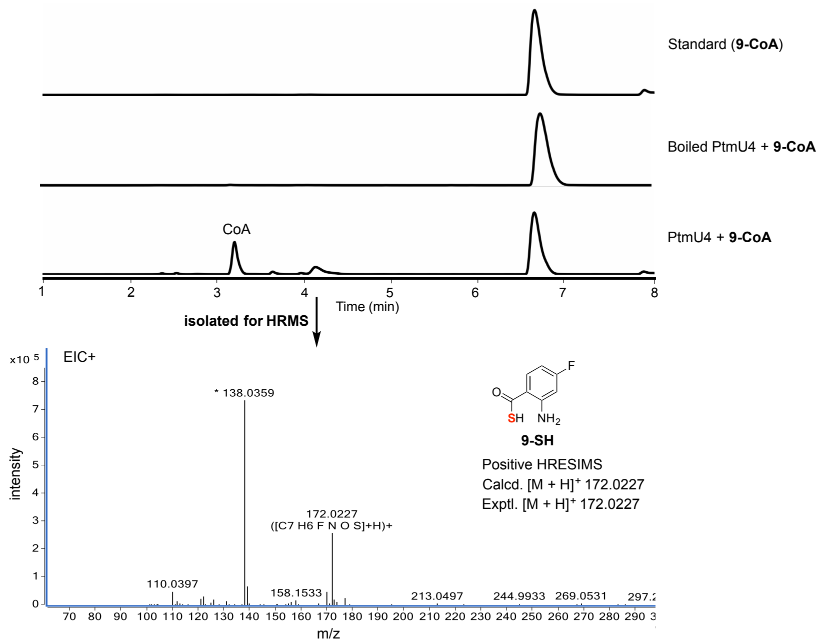

e

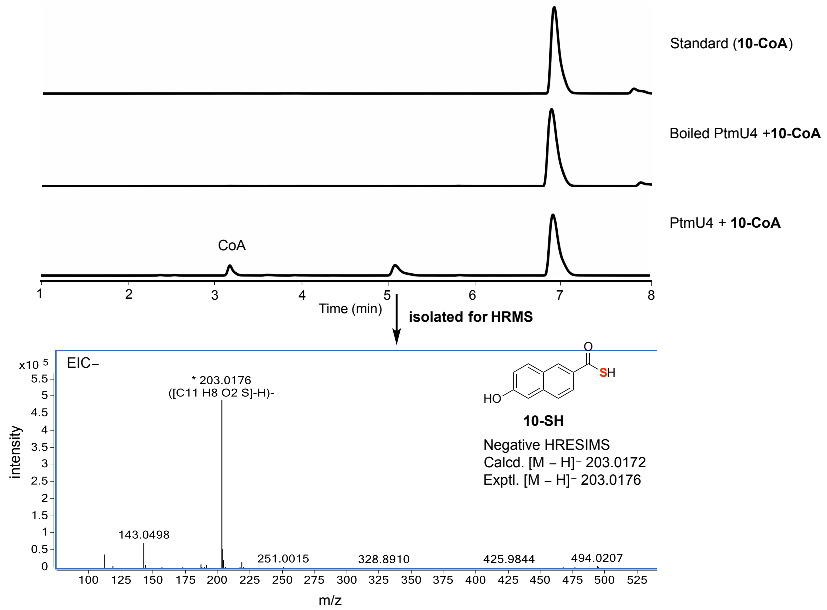

Supplementary Figure 48. HPLC chromatograms and HRESIMS spectra of PtmU4 enzyme reactions. The UV detection was at λ260 nm. New peaks from the PtmU4 enzyme reactions were isolated and injected to HRESIMS spectrometer. 3-amino-4-hydroxythiobenzoic acid (**6-SH**); 3-amino-4-chlorothiobenzoic acid (**7-SH**); 2-amino-3-hydroxythiobenzoic acid (**8-SH**); 2-amino-4-fluorothiobenzoic acid (**9-SH**); 6-hydroxy-2-naphthalenecarboxylic acid (**10-SH**).

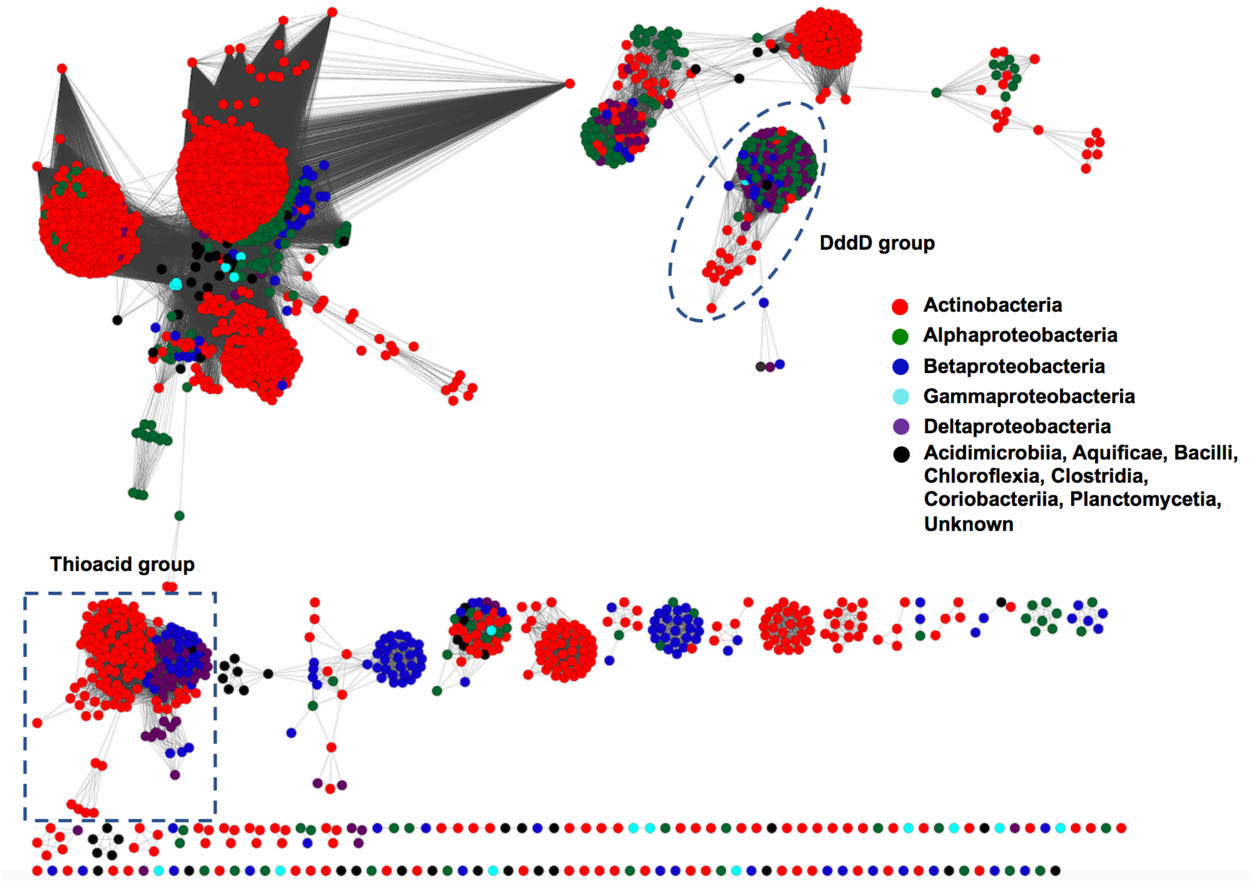

Supplementary Figure 49. Broad distribution of thiocarboxylic acid-containing natural products in nature. **a**, Sequence similarity network (SSN) of the two-domain type III CoA-transferases in bacteria. The SSN was generated using the online Enzyme Function Initiative-Enzyme Similarity Tool (EFI-EST)[24] and visualized in Cytoscape with a BLAST e-value threshold of 10^{-80} (median 40% sequence identity over 500 residues). Each node represents protein sequences sharing 100% sequence identity. Colors represent different classes in bacteria. The cluster containing DddD is circled; the predicted thiocarboxylic acid biosynthesis related type III CoA-transferases are boxed.

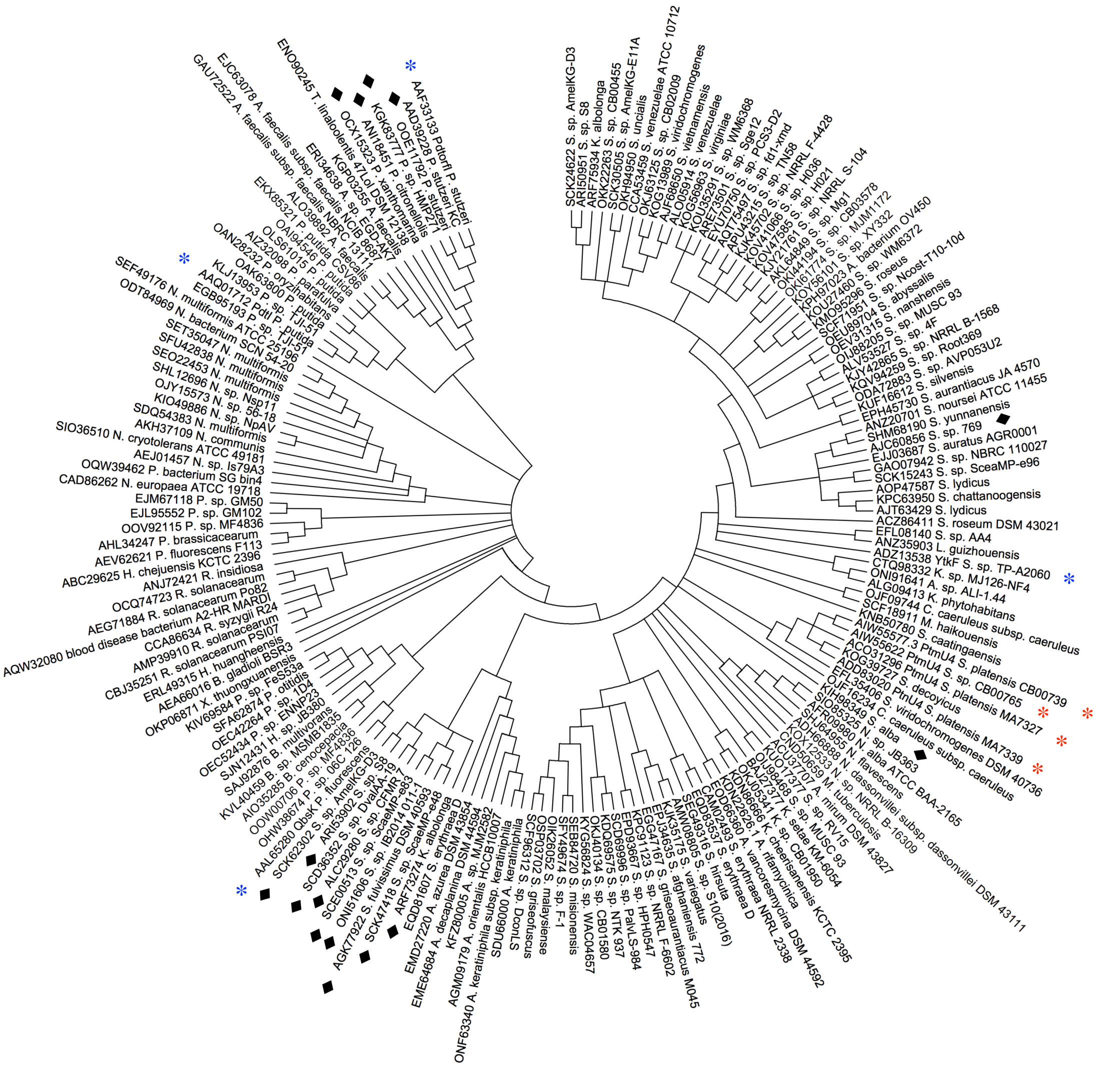

Supplementary Figure 50. Phylogenetic analysis of the "Thioacid" group. The protein sequences used in the phylogenetic tree include all sequences from the "Thioacid" group in the SSN (Supplementary Fig. 49). We analyzed a 21-gene region (10 genes each upstream and downstream of the *ptmU4* homologue) surrounding the *ptmU4* homologue in the genome of each strain, leading to the determination that 160 of the 175 homologues are encoded in genetic proximity (≤2 genes apart) to homologues of PtmA3. The remaining 15 homologues are indicated with a black diamond. PtmU4/PtnU4 are indicated with red asterisks, homologues of PtmU4 discussed in this study are indicated with black asterisks. The sequence identities of the PtmA3 homologues, not including PtmA3 proteins from *S. platensis*, range from 15–57%. The accession numbers of all PtmU4 and PtmA3 homologues used in this figure were summarized in Supplementary Table 7. The sequence alignment was generated as described above.

Supplementary References

1. Dong, L.-B., Rudolf, J. D. & Shen, B. Antibacterial sulfur-containing platensimycin and platencin congeners from *Streptomyces platensis* SB12029. *Bioorg. Med. Chem.* **24**, 6348-6353 (2016).
2. Louis-Jeune, C., Andrade-Navarro, M. A. & Perez-Iratxeta, C. Prediction of protein secondary structure from circular dichroism using theoretically derived spectra. *Proteins: Struct., Funct., Bioinf.* **80**, 374-381 (2012).
3. Wiegand, I., Hilpert, K. & Hancock, R. E. W. Agar and broth dilution methods to determine the minimal inhibitory concentration (MIC) of antimicrobial substances. *Nat. Protoc.* **3**, 163-175 (2008).
4. Kumar, S., Stecher, G. & Tamura, K. MEGA7: molecular evolutionary genetics analysis version 7.0 for bigger datasets. *Mol. Biol. Evol.* **33**, 1870-1874 (2016).
5. Hindra *et al.* Strain prioritization for natural product discovery by a high-throughput real-time PCR method. *J. Nat. Prod.* **77**, 2296-2303 (2014).
6. Gust, B., Challis, G. L., Fowler, K., Kieser, T. & Chater, K. F. PCR-targeted *Streptomyces* gene replacement identifies a protein domain needed for biosynthesis of the sesquiterpene soil odor geosmin. *Proc. Natl. Acad. Sci. U. S. A.* **100**, 1541-1546 (2003).
7. MacNeil, D. J. *et al.* Analysis of *Streptomyces avermitilis* genes required for avermectin biosynthesis utilizing a novel integration vector. *Gene* **111**, 61-68 (1992).
8. Rudolf, J. D., Dong, L.-B., Huang, T. & Shen, B. A genetically amenable platensimycin- and platencin-overproducer as a platform for biosynthetic explorations: a showcase of PtmO4, a long-chain acyl-CoA dehydrogenase. *Mol. BioSyst.* **11**, 2717-2726 (2015).
9. Chater, K. F. & Wilde, L. C. *Streptomyces albus* G mutants defective in the *Sal*GI restriction-modification system. *J. Gen. Microbiol.* **116**, 323-334 (1980).
10. Ziermann, R. & Betlach, M. C. Recombinant polyketide synthesis in *Streptomyces*: engineering of improved host strains. *BioTechniques* **26**, 106-110 (1999).
11. Komatsu, M. *et al.* Engineered *Streptomyces avermitilis* host for heterologous expression of biosynthetic gene cluster for secondary metabolites. *ACS Synth. Biol.* **2**, 384-396 (2013).
12. Gomez-Escribano, J. P. & Bibb, M. J. Engineering *Streptomyces coelicolor* for heterologous expression of secondary metabolite gene clusters. *Microb. Biotechnol.* **4**, 207-215 (2011).

13. Bierman, M. *et al.* Plasmid cloning vectors for the conjugal transfer of DNA from *Escherichia coli* to *Streptomyces* spp. *Gene* **116**, 43-49 (1992).
14. Doumith, M. *et al.* Analysis of genes involved in 6-deoxyhexose biosynthesis and transfer in *Saccharopolyspora erythraea*. *Mol. Gen. Genet.* **264**, 477-485 (2000).
15. Lohman, J. R., Bingman, C. A., Phillips, G. N. & Shen, B. Structure of the bifunctional acyltransferase/decarboxylase LnmK from the leinamycin biosynthetic pathway revealing novel activity for a double-hot-dog fold. *Biochemistry* **52**, 902-911 (2013).
16. Huang, W. *et al.* Characterization of yatakemycin gene cluster revealing a radical *S*-adenosylmethionine dependent methyltransferase and highlighting spirocyclopropane biosynthesis. *J. Am. Chem. Soc.* **134**, 8831-8840 (2012).
17. Rudolf, J. D., Dong, L.-B., Manoogian, K. & Shen, B. Biosynthetic origin of the ether ring in platensimycin. *J. Am. Chem. Soc.* **138**, 16711-16721 (2016).
18. Yu, Z. *et al.* Engineering of *Streptomyces platensis* MA7339 for overproduction of platencin and congeners. *Org. Lett.* **12**, 1744-1747 (2010).
19. Edgar, R. C. MUSCLE: multiple sequence alignment with high accuracy and high throughput. *Nucleic Acids Res.* **32**, 1792-1797 (2004).
20. Robert, X. & Gouet, P. Deciphering key features in protein structures with the new ENDscript server. *Nucleic Acids Res.* **42**, W320-W324 (2014).
21. Alcolombri, U., Laurino, P., Lara-Astiaso, P., Vardi, A. & Tawfik, D. S. DddD is a CoA-transferase/lyase producing dimethyl sulfide in the marine environment. *Biochemistry* **53**, 5473-5475 (2014).
22. Todd, J. D. *et al.* Structural and regulatory genes required to make the gas dimethyl sulfide in bacteria. *Science* **315**, 666-669 (2007).
23. Berthold, C. L., Toyota, C. G., Richards, N. G. J. & Lindqvist, Y. Reinvestigation of the catalytic mechanism of formyl-CoA transferase, a class III CoA-transferase. *J. Biol. Chem.* **283**, 6519-6529 (2008).
24. Gerlt, J. A. *et al.* Enzyme function initiative-enzyme similarity tool (EFI-EST): A web tool for generating protein sequence similarity networks. *Biochim. Biophys. Acta, Proteins Proteomics* **1854**, 1019-1037 (2015).
