# Supplementary material for: Biosynthesis of thiocarboxylic acid-containing natural products
Source: Nat Commun. 2018 Jun 18;9:2362. doi: 10.1038/s41467-018-04747-y (PMC6006322; doi:10.1038/s41467-018-04747-y)
Supplement: Supplementary file 2 — Descriptions of Additional Supplementary Files [file 41467_2018_4747_MOESM2_ESM.pdf]

Descriptions of Additional Supplementary Files

File Name: Supplementary Data 1
Description: Primers used in this study

File Name: Supplementary Data 2
Description: Accession numbers for the PtmU4 homologous proteins in the thioacid group in Supplementary Figure 49 and their PtmA3 homologues in genetic proximity.

File Name: Supplementary Data 3
Description: Cartesian coordinates and energies for molecules used in density functional theory calculations. All of the Cartesian coordinates correspond to M06-2X/6-311+G(d,p)-optimized geometries. Thermodynamic parameters are given in hartrees.
